# Supplementary material for: Prevalence of human infection with respiratory adenovirus in China: A systematic review and meta-analysis
Source: PLoS Negl Trop Dis. 2023 Feb 22;17(2):e0011151. doi: 10.1371/journal.pntd.0011151 (PMC9987798; doi:10.1371/journal.pntd.0011151)

S1 text

Supplement to: Prevalence of human infection with respiratory adenovirus in China: a systematic review and meta-analysis

# Table of Contents

Table A: Search Syntax 2

Table B: Criteria applied for abstract screening 3

Table C: Criteria applied for full text review of articles 4

Table D: Data extracted for each article included in the review 5

Table E: Article introducing adenovirus infection typing in China 6

Table F: Attack rate with 95% CI by meta-analysis of outbreak events 7

Table G: Detection rate with 95% CI by meta-analysis for the data of surveillance 8

Table H: Attack rate with 95% CI of figure 3A 9

Table I: Attack rate with 95% CI of figure 3B 10

Table J: Detection rate with 95% CI of figure 3C 11

Table K: Attack rate with 95% CI of figure 3D 12

Table L: Proportion with 95% CI of figure 5A 13

Table M: Proportions with 95% CI of figure 5B 14

Fig A: Distribution of adenovirus typing in different climatic regions of China. 15

# Table A: Search Syntax

| **Database** | **Query** |
| --- | --- |
| Pubmed | ('HAdV' OR 'adenovirus' [Title/Abstract]) AND ('respiratory' [Title/Abstract] OR 'pneumonia' [Title/Abstract]), AND ('China' OR 'the mainland of China' OR 'Chinese mainland' OR 'Taiwan' OR 'Hong Kong' OR 'Macau' OR 'Macao' [Title/Abstract]) |
| CNKI | (篇关摘%腺病毒) AND (篇关摘%呼吸道) AND发表时间: 2009-2021 |
| Wanfang | (题名或关键词:(腺病毒) and 题名或关键词:(呼吸道)) and Date:2009-2021 |
| VIP | 题名或关键词=腺病毒 并且 题名或关键词=呼吸道 并且 年份=2009-2021 |

# Table B: Criteria applied for abstract screening

| **Criteria** | **Guidance** | **Outcome** |
| --- | --- | --- |
| #1: Respiratory adenovirus | Does the Title/Abstract relate to respiratory adenovirus? | If Yes, remain and evaluate #2.  If No, exclude. |
| #2: Year | Does the year of publication period between 2009-2021? | If Yes, remain and evaluate #3.  If No, exclude. |
| #3: Human infection | Does the Title/Abstract refer the disease which are in human infection? | If Yes, remain and evaluate #4.  If No, exclude. |
| #4. Other types of papers | Does the Title/Abstract refer the article which is drug, vaccine trials, mechanism studies, animal experiments or reviews for HAdV? | If No, remain for full text review.  If Yes, exclude. |

# Table C: Criteria applied for full text review of articles

| **Criteria** | **Guidance** | **Outcome** |
| --- | --- | --- |
| #1: study design | Does the study is designed surveillance studies with sampling size <100 for laboratory test or HAdV positive detection <10 ? | If Yes, remain and evaluate #2.  If No, exclude. |
| #2: Not imported | Does the Title/Abstract refer the diseases which are not imported? | If Yes, remain and evaluate #3.  If No, exclude. |
| #3: Information | Does the article have information about methods of laboratory diagnosis, specimens tested ? | If Yes, remain and evaluate #4.  If No, exclude. |
| #4: Testing | Does the article refer the specific detection methods?  The test method is PCR, IgM, ELISA test, the test specimen is respiratory secretions or serum. | If Yes, remain and evaluate #5.  If No, exclude. |
| #5: Study period | The study period is 2009-2020 | If Yes, remain for data extracting.  If No, exclude |

# Table D: Data extracted for each article included in the review

| **Variable ID** | **Criteria** | **Guidance** |
| --- | --- | --- |
| 1 | Reference ID | Unique identifier assigned to an article. |
| 2 | Literature-related information | Author, publish year, province of study, start and end dates, name(s) of healthcare facility. |
| 3 | Study design | Seroprevalence study, outbreak investigation. |
| 4 | Setting | School/daycare outbreaks, healthcare comprised of hospitals and long-term care facilities, military camps, swimming pools. |
| 5 | The patients related information | Age group, patient population, mean or median age and proportion of female patients. |
| 6 | Laboratory test | Test methods applied, e.g., molecular, serological, type of HAdV. |
| 7 | Sample information | Sample size, absolute number or rate of positive detection. |
| 8 | Clinical information | Proportion of clinical symptoms or syndromes of patients if reported. |
| 9 | Additional information | Attack rate, number of primary cases and number of secondary cases. |

# Table E: Article introducing adenovirus infection typing in China

| **Type** | **Reference ID** |
| --- | --- |
| HAdV-1 | 11, 12, 13, 76, 101, 122, 126, 247, 248, 316, 341, 345, 363, 376, 377, 387, 395, 545, 576, 639, 644, 675, 776, 812, 817, 819, 827, 828, 831, 833, 835, 838, 839, 840, 842, 861, 874, 912, 944 |
| HAdV-2 | 11, 12, 13, 33, 51, 76, 101, 107, 122, 126, 157, 247, 248, 316, 341, 345, 363, 376, 377, 387, 395, 545, 553, 576, 639, 644, 675, 709, 765, 776, 812, 817, 827, 828, 831, 833, 835, 838, 839, 840, 842, 912, 944 |
| HAdV-3 | 11, 12, 13, 33, 51, 76, 101, 105, 107, 118, 122, 126, 137, 153, 157, 214, 218, 219, 220, 225, 247, 248, 285, 316, 341, 345, 363, 376, 377, 387, 395, 408, 472, 545, 553, 576, 625, 639, 644, 657, 675, 709, 765, 776, 779, 803, 812, 817, 818819, 827, 828, 831, 833, 835, 836, 838, 839, 840, 841, 842, 861, 863, 874, 885, 891, 912, 944 |
| HAdV-4 | 6, 12, 13, 33, 76, 101, 118, 122, 126, 153, 214, 228, 229, 247, 285, 316, 341, 345, 363, 545, 549, 576, 588, 644, 665, 667, 675, 677, 812, 817, 819, 828, 835, 838, 840, 842, 861, 874, 944 |
| HAdV-5 | 11, 12, 13, 101, 107, 118, 122, 126, 153, 247, 248, 316, 345, 377, 395, 545, 639, 644, 675, 765, 776, 812, 817, 819, 827, 828, 833, 835, 838, 839, 840, 842, 874, 912, 944 |
| HAdV-6 | 12, 13, 33, 101, 122, 126, 153, 247, 248, 316, 363, 376, 545, 639, 644, 812, 838, 839, 840, 842, 944 |
| HAdV-7 | 8, 11, 12, 13, 33, 51, 76, 101, 105, 107, 118, 122, 126, 137, 157, 205, 211, 212, 213, 215, 221, 223, 231, 235, 238, 247, 248, 284, 285, 286, 287, 316, 341, 345, 363, 376, 377, 393, 395, 458, 524, 545, 553, 576, 618, 619, 620, 622, 625, 639, 644, 675, 691, 709, 721, 729, 730, 731, 732, 776, 779, 803, 817, 818, 819, 820, 827, 828, 831, 833, 835, 836, 838, 840, 841, 842, 846, 847, 871, 874, 885, 891, 912, 922, 928, 933, 944, 949 |
| HAdV-11 | 101, 137, 625, 776 |
| HAdV-12 | 376 |
| HAdV-14 | 12, 13, 101, 122, 126, 345, 377, 641, 735, 765, 818, 835, 850, 885 |
| HAdV-21 | 76, 157, 545, 675, 828, 831, 835 |
| HAdV-31 | 341, 576, 644, 838 |
| HAdV-35 | 833 |
| HAdV-50 | 818 |
| HAdV-55 | 11, 12, 13, 101, 105, 107, 126, 157, 203, 204, 207, 234, 236, 237, 285, 458, 545, 591, 614, 621, 644, 675, 733, 810, 811, 818, 819, 823, 827, 828, 831, 838, 841, 845, 848, 849, 885, 891, 912, 928, 933 |
| HAdV-57 | 101, 122, 126, 395, 644, 835, 838, 842 |
| HAdV-104 | 817 |

# Table F: Attack rate with 95% CI by meta-analysis of outbreak events

|  | **Proportion by fixed effect % (95%CI)** | **Proportion by Random effect % (95%CI)** | **I^2^ (%)** | **Attack rate (%)** |
| --- | --- | --- | --- | --- |
| Number | 3.48 (3.36, 3.61) | 15.91 (13.85, 17.98) | 99.60 | 15.91 |
| Area |  |  |  |  |
| North | 9.25 (8.94, 9.55) | 19.01 (14.42, 23.60) | 99.50 | 19.01 |
| South | 2.39 (2.25, 2.52) | 13.53 (11.28, 15.77) | 99.60 | 13.53 |
| Season |  |  |  |  |
| Spring | 2.35 (2.15, 2.55) | 5.92 (4.47, 7.36) | 97.40 | 5.92 |
| Summer | 2.53 (2.16, 2.90) | 16.76 (12.47, 21.04) | 98.60 | 16.76 |
| Autumn | 1.23 (1.03, 1.44) | 5.71 (3.41, 8.01) | 98.30 | 5.71 |
| Winter | 11.65 (11.34, 11.95) | 22.65 (16.33, 28.96) | 99.70 | 22.65 |
| Age |  |  |  |  |
| Child | 12.05 (9.44, 14.65) | 12.78 (7.95, 17.62) | 64.30 | 12.78 |
| Adolescence | 1.77 (1.64, 1.89) | 6.52 (5.50, 7.55) | 97.80 | 6.52 |
| Adult | 21.70 (21.28, 22.12) | 23.56 (18.31, 28.80) | 99.30 | 23.56 |
| All-age groups | - | - | - | 25.29 |
| Setting |  |  |  |  |
| Military camps | 21.12 20.69, 21.56) | 23.55 (18.02, 29.07) | 99.40 | 23.55 |
| Hospital | 20.27 (15.39, 25.14) | 19.75 (8.64, 30.86) | 80.50 | 19.75 |
| Swimming pool | 12.98 (11.77, 14.19) | 22.47 (12.49, 32.45) | 98.20 | 22.47 |
| School | 1.81 (1.68, 1.94) | 6.19 (4.92, 7.46) | 98.70 | 6.19 |
| Type |  |  |  |  |
| HAdV-3 | 3.16 (2.84, 3.47) | 5.55 (2.63, 8.48) | 98.60 | 5.55 |
| HAdV-4 | 2.77 (2.17, 3.38) | 8.75 (0.00, 21.30) | 96.40 | 8.75 |
| HAdV-7 | 9.47 (9.10, 9.85) | 22.32 (14.78, 29.86) | 99.70 | 22.32 |
| HAdV-14 | - | - | - | 8.83 |
| HAdV-55 | 27.18 (26.38, 27.98) | 27.18 (19.16, 35.20) | 98.80 | 27.18 |

# Table G: Detection rate with 95% CI by meta-analysis for the data of surveillance

|  | **Positive rate by fixed effect % (95%CI)** | **Positive rate by Random effect % (95%CI)** | **I^2^ (%)** | **Detection rate (%)** |
| --- | --- | --- | --- | --- |
| Number | 1.22 (1.20, 1.23) | 4.21 (4.07, 4.34) | 99.10 | 4.21 |
| Area |  |  |  |  |
| North | 0.82 (0.80, 0.84) | 4.15 (3.95, 4.34) | 98.30 | 4.15 |
| South | 1.38 (1.36, 1.39) | 4.13 (3.95, 4.31) | 99.20 | 4.13 |
| Unknown | 4.07 (3.90, 4.24) | 6.85 (5.07, 8.63) | 98.50 | 6.85 |
| Season |  |  |  |  |
| Spring | 1.63 (1.52, 1.74) | 5.21 (4.35, 6.07) | 97.40 | 5.21 |
| Summer | 1.57 (1.45, 1.69) | 4.64 (3.82, 5.47) | 96.90 | 4.64 |
| Autumn | 1.35 (1.24, 1.46) | 3.62 (2.95, 4.29) | 95.60 | 3.62 |
| Winter | 1.11 (1.02, 1.20) | 3.54 (2.83, 4.24) | 97.70 | 3.54 |
| Age |  |  |  |  |
| Children | 0.93 (0.90, 0.97) | 4.04 (3.76, 4.31) | 97.90 | 4.04 |
| Adolescence | 1.00 (0.00.85, 1.14) | 4.45 (2.55, 6.36) | 98.30 | 4.45 |
| Adult | 0.72 (0.63, 0.82) | 3.44 (2.76, 4.12) | 97.50 | 3.44 |
| The elderly | 0.06 (0.01, 0.11) | 2.81 (1.84, 3.78) | 96.80 | 2.81 |
| All-age groups | 1.38 (1.36, 1.39) | 4.29 (4.12, 4.45) | 99.20 | 4.29 |
| Setting |  |  |  |  |
| Hospital | 1.22 (1.20, 1.23) | 4.21 (4.07, 4.34) | 99.10 | 4.21 |
| Type |  |  |  |  |
| HAdV-1 | 4.55 (4.05, 5.05) | 6.70 (5.39, 8.01) | 80.60 | 6.70 |
| HAdV-2 | 2.36 (2.04, 2.68) | 8.90 (7.31, 10.50) | 94.20 | 8.90 |
| HAdV-3 | 39.56 (38.99, 40.12) | 32.73 (22.13, 3.34) | 97.90 | 31.38 |
| HAdV-4 | 1.12 (0.84, 1.40) | 2.07 (1.41, 2.74) | 69.40 | 2.07 |
| HAdV-5 | 2.47 (2.09, 2.85) | 3.55 (2.78, 4.32) | 67.80 | 3.61 |
| HAdV-6 | 1.26 (0.87, 1.64) | 1.97 (1.23, 2.70) | 58.20 | 1.97 |
| HAdV-7 | 32.93 (32.42, 33.44) | 27.48 (17.04, 37.91) | 99.70 | 7.48 |
| HAdV-11 | 5.66 (4.24, 7.08) | 16.14 (1.89, 30.40) | 98.70 | 16.14 |
| HAdV-12 | - | - | - | 0.84 |
| HAdV-14 | 0.74 (0.32, 1.15) | 2.01 (0.92, 3.09) | 68.40 | 2.01 |
| HAdV-21 | 0.36 (0.12, 0.59) | 0.87 (0.17, 1.57) | 73.80 | 0.87 |
| HAdV-31 | 0.33 (0.00, 0.73) | 0.33 (0.00, 0.73) | 0.00 | 0.33 |
| HAdV-35 | - | - | - | 1.39 |
| HAdV-50 | - | - | - | 4.17 |
| HAdV-55 | 0.78 (0.56, 1.00) | 4.70 (3.40, 6.00) | 95.80 | 4.70 |
| HAdV-57 | 1.01 (0.51, 1.50) | 1.01 (0.51, 1.50) | 0.00 | 1.01 |
| HAdV-104 | - | - | - | 0.81 |

# Table H: Attack rate with 95% CI of figure 3A

| **Month** | **Proportion by fixed effect % (95%CI)** | **Proportion by Random effect % (95%CI)** | **I^2^ (%)** | **Attack rate (%)** |
| --- | --- | --- | --- | --- |
| North | 9.25 (8.94, 9.55) | 19.01 (14.42, 23.60) | 99.50 | 19.01 |
| 1 | 16.97 (16.26, 17.67) | 17.61 (14.39, 20.84) | 93.60 | 17.61 |
| 2 | 19.56 (18.70, 20.42) | 20.23 (7.78, 32.68) | 99.50 | 20.23 |
| 4 | 1.43 (0.86, 1.99) | 4.83 (0.00, 1247) | 97.10 | 4.83 |
| 7 | - | - | - | 58.22 |
| 8 | - | - | - | 24.00 |
| 10 | 3.17 (2.50, 3.84) | 9.54 (0.00, 24.23) | 99.20 | 9.54 |
| 11 | - | - | - | 3.83 |
| 12 | 21.95 (20.93, 22.97) | 25.20 (5.68, 44.72) | 99.70 | 25.20 |
| South | 2.39 (2.25, 2.52) | 13.53 (11.28, 15.77) | 99.60 | 13.53 |
| 1 | - | - | - | 38.10 |
| 2 | 32.61 (31.30, 33.92) | 32.61 (31.30, 33.92) | 0.00 | 32.61 |
| 4 | 3.01 (2.10, 3.91) | 8.89 (0.00, 21.73) | 96.10 | 8.89 |
| 5 | 2.45 (2.23, 2.67) | 6.15 (4.34, 7.96) | 97.90 | 6.15 |
| 6 | 1.88 (1.49, 2.26) | 4.94 (2.15, 7.73) | 96.60 | 4.94 |
| 7 | - | - | - | 27.83 |
| 8 | 8.25 (6.73, 9.77) | 11.42 (5.30, 17.55) | 91.10 | 11.42 |
| 10 | - | - | - | 10.69 |
| 11 | 0.85 (0.63, 1.07) | 1.80 (0.00, 4.37) | 98.40 | 1.80 |
| 12 | 3.28 (2.86, 3.69) | 23.27 (0.00, 50.60) | 99.90 | 23.27 |

# Table I: Attack rate with 95% CI of figure 3B

| **Month** | **Proportion by fixed effect % (95%CI)** | **Proportion by Random effect % (95%CI)** | **I^2^ (%)** | **Attack rate (%)** |
| --- | --- | --- | --- | --- |
| Hospital | 20.27 (15.39, 25.14) | 19.75 (8.64, 30.86) | 80.50 | 19.75 |
| 1 | - | - | - | 13.95 |
| 6 | - | - | - | 25.29 |
| Military camps | 21.12 (20.69, 21.56) | 23.55 (18.02, 29.07) | 99.40 | 23.55 |
| 1 | 18.59 (17.91, 19.27) | 21.08 (16.14, 26.03) | 97.70 | 21.08 |
| 2 | 21.83 (21.05, 22.61) | 21.98 (10.98, 32.97) | 99.50 | 21.98 |
| 10 | 17.08 (14.59, 19.57) | 17.08 (14.59, 19.57) | - | 17.08 |
| 12 | 24.77 (23.91, 25.62) | 31.76 (11.12, 52.40) | 99.80 | 31.76 |
| School | 1.81 (1.68, 1.94) | 6.19 (4.92, 7.46) | 98.70 | 6.19 |
| 2 | 32.79 (30.94, 34.65) | 32.79 (30.94, 34.65) | - | 32.79 |
| 4 | 1.87 (1.39, 2.35) | 5.90 (2.76, 9.04) | 95.60 | 5.90 |
| 5 | 2.33 (2.11, 2.56) | 4.86 (3.21, 6.51) | 97.40 | 4.86 |
| 6 | 1.80 (1.41, 2.18) | 1.92 (0.67, 3.16) | 90.00 | 1.92 |
| 8 | - | - | - | 24.00 |
| 10 | 2.51 (1.83, 3.18) | 6.25 (0.00, 14.68) | 96.50 | 6.25 |
| 11 | - | - | - | 2.45 |
| 12 | 1.17 (0.73, 1.60) | 8.01 (0.00, 22.06) | 97.10 | 8.01 |
| Swimming pool | 12.98 (11.77, 14.19) | 22.47 (12.49, 32.45) | 98.20 | 22.47 |
| 5 | - | - | - | 14.89 |
| 7 | 39.24 (35.29, 43.19) | 42.96 (13.17, 72.74) | 98.10 | 42.96 |
| 8 | 8.25 (6.73, 9.77) | 11.42 (5.30, 17.55) | 91.10 | 11.42 |

# Table J: Detection rate with 95% CI of figure 3C

| **Month** | **Proportion by fixed effect % (95%CI)** | **Proportion by Random effect % (95%CI)** | **I^2^ (%)** | **Detection rate (%)** |
| --- | --- | --- | --- | --- |
| North |  |  |  |  |
| 1 | 1.55 (1.21, 1.89) | 4.41 (2.80, 6.01) | 91.80 | 4.41 |
| 2 | 1.53 (1.12, 1.95) | 3.89 (2.28, 5.49) | 87.10 | 3.89 |
| 3 | 2.37 (1.82, 2.93) | 4.25 (2.54, 5.97) | 82.10 | 4.25 |
| 4 | 3.45 (2.76, 4.15) | 7.71 (4.77, 10.66) | 91.40 | 7.71 |
| 5 | 2.74 (2.20, 3.28) | 6.78 (4.04, 9.53) | 94.20 | 6.78 |
| 6 | 2.20 (1.70, 2.71) | 6.06 (3.70, 8.42) | 93.00 | 6.06 |
| 7 | 2.01 (1.50, 2.53) | 4.11 (2.36, 5.85) | 83.30 | 4.11 |
| 8 | 2.71 (2.08, 3.35) | 3.03 (1.87, 4.20) | 52.50 | 3.03 |
| 9 | 4.30 (3.42, 5.18) | 5.40 (3.28, 7.52) | 73.60 | 5.40 |
| 10 | 4.17 (3.31, 5.03) | 4.13 (2.41, 5.85) | 61.10 | 4.13 |
| 11 | 2.03 (1.54, 2.53) | 4.85 (2.63, 7.06) | 89.10 | 4.85 |
| 12 | 1.37 (1.03, 1.71) | 3.31 (01.96, 4.66) | 89.40 | 3.31 |
| South |  |  |  |  |
| 1 | 1.84 (1.61, 2.07) | 3.36 (2.22, 4.50) | 94.50 | 3.36 |
| 2 | 1.72 (1.47, 1.96) | 3.16 (2.02, 4.29) | 93.40 | 3.16 |
| 3 | 1.66 (1.44, 1.88) | 3.91 (2.74, 5.08) | 94.00 | 3.91 |
| 4 | 2.14 (1.86, 2.42) | 4.45 (3.13, 5.77) | 93.30 | 4.45 |
| 5 | 2.41 (2.12, 2.70) | 4.41 (3.15, 5.68) | 92.20 | 4.41 |
| 6 | 1.67 (1.43, 1.90) | 4.93 (3.57, 6.29) | 94.40 | 4.93 |
| 7 | 2.11 (1.83, 2.39) | 4.64 (3.27, 6.01) | 93.30 | 4.64 |
| 8 | 1.89 (1.61, 2.18) | 3.99 (2.71, 5.26) | 91.70 | 3.99 |
| 9 | 1.21 (0.98, 1.45) | 2.81 (1.91, 3.72) | 87.40 | 2.81 |
| 10 | 1.22 (1.00, 1.44) | 2.68 (1.79, 3.56) | 89.60 | 2.68 |
| 11 | 1.44 (1.21, 1.67) | 3.58 (2.56, 4.60) | 91.80 | 3.58 |
| 12 | 1.68 (1.45, 1.92) | 3.58 (2.29, 4.86) | 95.50 | 3.58 |

# Table K: Attack rate with 95% CI of figure 3D

| **Month** | **Proportion by fixed effect % (95%CI)** | **Proportion by Random effect % (95%CI)** | **I^2^ (%)** | **Attack rate (%)** |
| --- | --- | --- | --- | --- |
| Adult | 21.70 (21.28, 22.12) | 23.56 (18.31, 28.80) | 99.30 | 23.56 |
| 1 | 18.55 (17.87, 19.23) | 20.48 (15.77, 25.19) | 97.50 | 20.48 |
| 2 | 23.47 (22.75, 24.18) | 23.33 (13.53, 33.13) | 99.50 | 23.33 |
| 10 | - | - | - | 17.08 |
| 12 | 24.77 (23.91, 25.62) | 31.76 (11.12, 52.40) | 99.80 | 31.76 |
| Children | 12.05 (9.44, 14.65) | 12.78 (7.95, 17.62) | 64.30 | 12.78 |
| 4 | - | - | - | 15.69 |
| 10 | - | - | - | 10.69 |
| Teenager | 1.77 (1.64, 1.89) | 6.52 (5.50, 7.55) | 97.80 | 6.52 |
| 4 | 1.74 (1.26, 2.22) | 3.76 (1.08, 6.44) | 94.90 | 3.76 |
| 5 | 2.45 (2.23, 2.67) | 6.15 (4.34, 7.96) | 97.90 | 6.15 |
| 6 | 1.80 (1.41, 2.18) | 1.92 (0.67, 3.16) | 90.00 | 1.92 |
| 7 | 39.24 (35.29, 43.19) | 42.96 (13.17, 72.74) | 98.10 | 42.96 |
| 8 | 8.51 (7.00, 10.02) | 13.34 (7.16, 19.51) | 89.70 | 13.34 |
| 10 | - | - | - | 2.09 |
| 11 | 0.98 (0.77, 1.20) | 2.45 (0.22, 4.68) | 97.90 | 2.45 |
| 12 | 1.17 (0.73, 1.60) | 8.01 (0.00, 22.06) | 97.10 | 8.01 |

# Table L: Proportion with 95% CI of figure 5A

|  | **Proportion by fixed effect % (95%CI)** | **Proportion by Random effect % (95%CI)** | **I^2^ (%)** | **Proportion (%)** |
| --- | --- | --- | --- | --- |
| Fever | 99.93 (99.82, 100.00) | 98.34 (97.68, 99.00) | 95.70 | 98.34 |
| Children | 99.93 (99.86, 100.00) | 97.88 (97.36, 98.41) | 94.90 | 97.88 |
| Teenager | 99.95 (99.85, 100.00) | 81.39 (77.36, 85.43) | 97.40 | 81.39 |
| Adult | 99.68 (99.05, 100.00) | 98.63 (97.16, 100.00) | 72.70 | 98.63 |
| Cough | 80.36 (79.70, 81.02) | 66.28 (60.65, 71.90) | 98.50 | 66.28 |
| Children | 87.48 (86.34, 88.61) | 76.47 (68.04, 84.90) | 97.30 | 76.47 |
| Teenager | 41.58 (38.74, 44.41) | 47.90 (34.23, 61.56) | 95.50 | 47.90 |
| Adult | 79.87 (79.03, 80.71) | 72.40 (64.86, 79.94) | 98.70 | 72.40 |
| Expectoration | 59.36 (58.17, 60.55) | 52.74 (41.49, 63.99) | 98.80 | 52.74 |
| Children | 60.83 (59.59, 62.07) | 56.28 (43.41, 69.16) | 99.00 | 56.28 |
| Teenager | 49.35 (43.77, 54.93) | 49.35 (43.77, 54.93) | 0.00 | 49.35 |
| Adult | 31.79 (25.17, 38.40) | 31.19 (21.06, 41.32) | 54.60 | 31.19 |
| Respiratory failure | 2.47 (1.57, 3.37) | 5.68 (2.51, 8.86) | 89.40 | 5.68 |
| Children | 5.55 (3.45, 7.65) | 11.05 (0.66, 21.43) | 93.60 | 11.05 |
| Adult | 1.78 (0.78, 2.77) | 1.78 (0.78, 2.77) | 0.00 | 1.78 |
| Breathing difficulties | 15.13 (14.17, 16.09) | 22.57 (14.28, 30.85) | 98.40 | 22.57 |
| Children | 5.71 (1.72, 9.71) | 5.71 (1.72, 9.71) | 0.00 | 34.69 |
| Teenager | 29.74 (28.13, 31.34) | 34.69 (21.59, 47.80) | 98.10 | 5.71 |
| Adult | 7.07 (5.81, 8.33) | 13.92 (6.59, 21.25) | 95.10 | 13.92 |
| Tonsil enlargement | 96.43 (96.14, 96.71) | 63.64 (52.01, 75.28) | 99.80 | 63.64 |
| Children | 35.57 (33.15, 37.99) | 37.77 (30.41, 45.13) | 57.90 | 37.77 |
| Teenager | 97.63 (97.34, 97.92) | 70.89 (55.92, 85.87) | 99.90 | 70.89 |
| Adult | 64.98 (62.15, 67.81) | 62.63 (39.28, 85.99) | 98.50 | 62.63 |
| Running nose | 12.20 (10.84, 13.56) | 22.51 (17.21, 27.82) | 92.10 | 22.51 |
| Children | 29.58 (25.37, 33.80) | 39.59 (19.08, 60.09) | 95.00 | 29.58 |
| Teenager | 19.62 (16.29, 22.96) | 27.22 (17.77, 36.67) | 85.10 | 19.62 |
| Adult | 8.05 (6.46, 9.64) | 10.04 (5.85, 14.22) | 83.10 | 8.05 |
| Vomiting | 3.22 (2.41, 4.03) | 11.35 (7.91, 14.79) | 89.10 | 11.35 |
| Children | - | - | -- | 25.00 |
| Teenager | 12.09 (9.60, 14.58) | 15.43 (10.53, 20.32) | 68.80 | 15.43 |
| Adult | 2.09 (1.23, 2.95) | 4.60 (0.64, 8.56) | 92.90 | 4.60 |
| Diarrhea | 5.72 (5.17, 6.27) | 10.16 (7.57, 12.76) | 94.70 | 10.16 |
| Children | 14.33 (9.41, 19.24) | 16.87 (5.94, 27.79) | 54.10 | 16.87 |
| Teenager | 3.55 (1.29, 5.81) | 3.55 (1.29, 5.81) | 0.00 | 3.55 |
| Adult | 5.74 (5.17, 6.31) | 11.16 (8.10, 14.21) | 95.90 | 11.16 |
| Pneumonia | 76.95 (76.51, 77.38) | 35.61 (19.40, 51.82) | 99.90 | 35.61 |
| Children | 99.07 (98.56, 99.59 | 66.56 (59.17, 73.96) | 99.30 | 66.56 |
| Teenager | 15.47 (11.71, 19.22) | 21.69 (11.94, 31.45) | 82.40 | 21.69 |
| Adult | 17.94 (17.08, 18.80) | 25.03 (9.42, 40.65) | 99.70 | 25.03 |

# Table M: Proportions with 95% CI of figure 5B

|  | **Proportion by fixed effect % (95%CI)** | **Proportion by Random effect % (95%CI)** | **I^2^ (%)** | **Proportion (%)** |
| --- | --- | --- | --- | --- |
| Fever | 99.96 (99.85, 100.00) | 99.58 (99.14, 100.00) | 85.00 | 99.82 |
| HAdV-7 | 99.97 (99.86, 100.00) | 99.82 (99.48,100.00) | 75.90 | 97.85 |
| HAdV-55 | 99.87 (99.30, 100.00) | 97.85 (95.45, 100.00) | 87.70 | 85.25 |
| HAdV-3 | 94.14 (90.61, 97.66) | 85.25 (55.93, 100.00) | 97.70 | 99.58 |
| Cough | 90.89 (86.59, 95.19) | 79.91 (52.35, 100.00) | 90.90 | 70.08 |
| HAdV-7 | 81.21 (80.31, 82.12) | 70.08 (61.34, 78.82) | 98.80 | 74.21 |
| HAdV-55 | 81.77 (80.78, 82.76) | 74.21 (63.99, 84.43) | 99.00 | 56.61 |
| HAdV-3 | 73.36 (70.69, 76.04) | 56.61 (28.01, 85.21) | 98.80 | 79.91 |
| Expectoration | 56.47 (54.84, 58.10) | 54.08 (38.42, 69..74) | 98.80 | 54.08 |
| HAdV-7 | 63.75 (61.86, 65.65) | 60.59 (43.44, 77.74) | 98.70 | 60.59 |
| HAdV-55 | 36.02 (32.84, 39.20) | 45.10 (13.84, 76.35) | 98.60 | 45.10 |
| Breathing difficulties | 13.18 (11.13, 15.22) | 21.60 (6.09, 37.12) | 97.80 | 21.60 |
| HAdV-7 | 13.24 (11.02, 15.46) | 26.80 (4.33, 49.26) | 98.70 | 26.80 |
| HAdV-3 | - | - | - | 14.96 |
| Tonsil enlargement | 97.57 (97.28, 97.85) | 68.43 (54.62, 82.23) | 99.90 | 68.43 |
| HAdV-7 | 97.36 (97.05, 97.66) | 67.31 (40.60, 94.03) | 99.90 | 67.31 |
| HAdV-55 | 99.64 (98.76, 100.00) | 96.91 (90.36, 100.00) | 91.00 | 96.91 |
| HAdV-3 | - | - | - | 20.59 |
| Running nose | 8.52 (6.95, 10.09) | 12.46 (7.84, 17.07) | 85.20 | 12.46 |
| HAdV-7 | 10.28 (8.26, 12.30) | 14.38 (7.63, 21.13) | 88.50 | 14.38 |
| HAdV-55 | 5.33 (2.79, 7.86) | 6.90 (1.70, 12.09) | 63.40 | 6.90 |
| HAdV-3 | - | - | - | 20.59 |
| Vomiting | 2.45 (1.60, 3.29) | 7.19 (3.30, 11.08) | 91.40 | 7.19 |
| HAdV-7 | 2.32 (1.45, 3.19) | 7.02 (2.55, 11.49) | 93.20 | 7.02 |
| HAdV-55 | - | - | - | 3.37 |
| HAdV-3 | - | - | - | 20.59 |
| Diarrhea | 4.41 (3.78, 5.03) | 9.22 (5.88, 12.56) | 95.50 | 9.22 |
| HAdV-7 | 5.02 (4.33, 5.70) | 10.90 (6.90, 14.91) | 95.90 | 10.90 |
| HAdV-55 | 1.50 (0.00, 3.00) | 1.50 (0.00, 3.00) | 0.00 | 1.50 |
| Pneumonia | 9.96 (9.07, 10.85) | 19.12 (14.11, 24.12) | 96.20 | 19.12 |
| HAdV-7 | 9.67 (8.77, 10.57) | 18.32 (12.87, 23.77) | 96.80 | 18.32 |
| HAdV-55 | 27.91 (18.43, 37.39) | 27.91 (18.43, 37.39) | 0.00 | 27.91 |
| HAdV-3 | - | - | - | 16.54 |

# Fig A: Distribution of adenovirus typing in different climatic regions of China.

Seven types of geographical regions were defined as Northeast China, North China, Inner Mongolia-Xinjiang, Qinghai-Tibet, Southwest China, Central China, and South China, according to the climatic and ecological characteristics. The pie chart represents the distribution of adenovirus types in different climatic regions. Pie chart I indicates the surveillance reports, and pie chart II indicates the outbreak investigations. No data has been reported on the adenovirus typing in Southwest China. The base layer of the map is publically available on the Resource and Environmental Sciences and Data Centre (https://www.resdc.cn/DOI/DOI.aspx?DOIID=122).


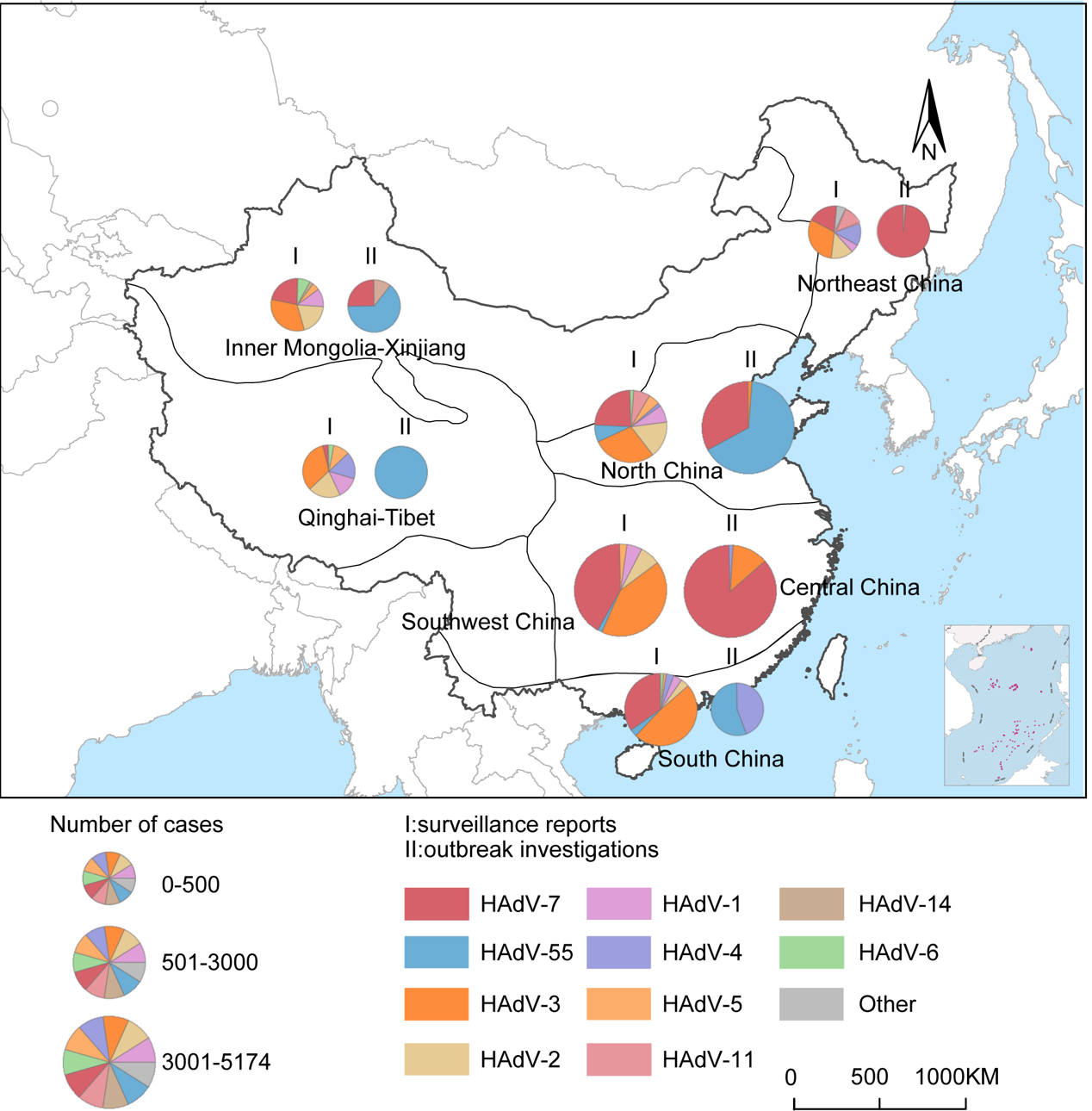

Supplement: S1 Text — (DOCX) [file pntd.0011151.s002.docx]
