# Supplementary material for: Prevalence of human infection with respiratory adenovirus in China: A systematic review and meta-analysis
Source: PLoS Negl Trop Dis. 2023 Feb 22;17(2):e0011151. doi: 10.1371/journal.pntd.0011151 (PMC9987798; doi:10.1371/journal.pntd.0011151)
Supplement: S2 Text — (DOCX) [file pntd.0011151.s003.docx]

**S2 Text**

Supplement to: Prevalence of human infection with respiratory adenovirus in China: a systematic review and meta-analysis

# Forest plots for meta-analysis

(A) Results of meta-analysis of Table 1:

(a1) outbreaks, (a2) outbreaks group by area, (a3) outbreaks group by season, (a4) outbreaks group by age, (a5) outbreaks group by setting, (a6) outbreaks group by type, (a7) surveillances, (a8) surveillances group by area, (a9) surveillances in spring, (a10) surveillances in summer, (a11) surveillances in autumn, (a12) surveillance in winter, (a13) surveillances group by age, (a14) surveillances in HAdV-1, (a15) surveillances in HAdV-2, (a16) surveillances in HAdV-3, (a17) surveillances in HAdV-4, (a18) surveillances in HAdV-5, (a19) surveillances in HAdV-6, (a20) surveillances in HAdV-7; (a21) surveillances in HAdV-11; (a22) surveillances in HAdV-14; (a23) surveillances in HAdV-21; (a24) surveillances in HAdV-31; (a25) surveillances in HAdV-55; (a26) surveillances in HAdV-57;

(B) Results of meta-analysis of figure 3:

(b1) north outbreaks group by month, (b2) south outbreaks group by month, (b3) January surveillance in north, (b4) February surveillance in north, (b5) March surveillance in north, (b6) April surveillance in north, (b7) May surveillance in north, (b8) June surveillance in north, (b9) July surveillance in north, (b10) August surveillance in north, (b11) September surveillance in north, (b12) October surveillance in north, (b13) November surveillance in north, (b14) December surveillance in north, (b15) January surveillance in south, (b16) February surveillance in south, (b17) March surveillance in south, (b18) April surveillance in south, (b19) May surveillance in south, (b20) June surveillance in south, (b21) July surveillance in south, (b22) August surveillance in south, (b23) September surveillance in south, (b24) October surveillance in south, (b25) November surveillance in south, (b26) December surveillance in south, (b27) meta-analysis of adult, (b28) meta-analysis of teenager, (b29) meta-analysis of military, (b30) meta-analysis of school, (b31) meta-analysis of swimming pool;

(C) Results of meta-analysis of figure 5:

(c1) fever group by age, (c2) fever group by type, (c3) cough group by age, (c4) cough group by type, (c5) expectoration group by age, (c6) expectoration group by type, (c7) respiratory failure group by age, (c8) breathing difficulties group by age, (c9) breathing difficulties group by type, (c10) tonsillar enlargement difficulties group by age, (c11) tonsillar enlargement difficulties group by type, (c12) running nose group by age, (c13) running nose group by type, (c14) diarrhea group by age, (c15) diarrhea group by type, (c16) vomiting group by age, (c17) vomiting group by type, (c18) pneumonia group by age, (c19) pneumonia group by type.

(A)

(a1)

(a2)

(a3)

(a4)

(a5)

(a6)

(a7)

(a8)

(a9)

(a10)

(a11)

(a12)

(a13)

(a14)

(a15)

(a16)

(a17)

(a18)

(a19)

(a20)

(a21)

(a22)

(a23)

(a24)

(a25)

(a26)

(B)

(b1)


(b2)

(b3)

(b4)

(b5)

(b6)

(b7)

(b8)

(b9)

(b10)

(b11)

(b12)

(b13)

(b14)

(b15)

(b16)

(b17)

(b18)

(b19)

(b20)

(b21)

(b22)

(b23)

(b24)

(b25)

(b26)

(b27)


(b28)

(b29)

(b30)

(b31)

C

c(1)

c(2)

c(3)

c(4)

c(5)

c(6)

c(7)

c(8)

c(9)

c(10)

c(11)

c(12)

c(13)

c(14)

c(15)

c(16)

c(17)


c(18)

c(19)
