# Supplementary material for: Prevalence of human infection with respiratory adenovirus in China: A systematic review and meta-analysis
Source: PLoS Negl Trop Dis. 2023 Feb 22;17(2):e0011151. doi: 10.1371/journal.pntd.0011151 (PMC9987798; doi:10.1371/journal.pntd.0011151)
Supplement: Attachment — Submitted filename: PNTD-D-22-01337_reviewer_revised.pdf [file pntd.0011151.s005.pdf]

PLOS Neglected Tropical Diseases

Human infection with respiratory adenovirus in China: a systematic review and meta-analysis

--Manuscript Draft--

**Manuscript Number:**	PNTD-D-22-01337
**Full Title:**	Human infection with respiratory adenovirus in China: a systematic review and meta-analysis
**Short Title:**	Human infection with respiratory adenovirus in China
**Article Type:**	Research Article
**Keywords:**	Human adenovirus; China; respiratory tract infections; Meta-analysis
**Abstract:**	Background<br><br>Human adenovirus (HAdV) is a major pathogen that causes respiratory tract infections (ARTI) and frequently associated with outbreaks. The HAdV prevalence and the predominant types ~~that were~~ responsible for ~~the~~ ARTI outbreaks ~~remained~~ obscure in China.<br><br>Methods<br><br>A systematic review was performed to retrieve literature that reported outbreaks or etiological surveillance of HAdV in China from January 2009 to March 2021. The genetic characteristics and epidemiological characteristics of HAdVs were investigated.<br><br>Results<br><br>A total of 950 articles (91 about outbreaks and 859 about etiological surveillance) meeting the selection criteria were included. Predominant HAdV types from etiological surveillance studies differed from those in outbreak events. Among the 70 outbreaks for which the HAdVs were typed, nearly half (45·71%) were caused by HAdV-7 with an overall attack rate of 22·32% after meta-analysis. Military camp and school were predominant settings of outbreaks with HAdV-55 and HAdV-7 respectively identified as the leading type and with significantly different seasonal pattern and attack rate. Among 859 hospital-based surveillance studies, positive detection rates of HAdV-3 (32·73%) and HAdV-7 (27·48%) were significantly higher than other types. Clinical manifestations mainly depended on HAdV types and patient age with significant discrepancy among them, and infection with HAdV-55 tends to develop pneumonia with a poorer prognosis, especially for children <5 years old.<br><br>Conclusions<br><br>This study improves the understanding of epidemiological and clinical features of HAdV infections and outbreaks with different types, and helps to inform future surveillance and control efforts in different settings.
**Additional Information:**	
**Question**	**Response**
**Government Employee**<br><br>Are you or any of the contributing authors an employee of the United States government?<br><br>Manuscripts authored by one or more US	No - No authors are employees of the U.S. government.

Powered by Editorial Manager® and ProduXion Manager® from Aries Systems Corporation

Government employees are not copyrighted, but are licensed under a CC0 Public Domain Dedication, which allows unlimited distribution and reuse of the article for any lawful purpose. This is a legal requirement for US Government employees.<br><br>This will be typeset if the manuscript is accepted for publication.	
**Financial Disclosure**<br><br>Enter a financial disclosure statement that describes the sources of funding for the work included in this submission and the role the funder(s) played. This includes grants and any commercial funding of the work or authors.<br><br>This statement will be typeset if the manuscript is accepted for publication.<br><br>Review the submission guidelines and the instructions link below for detailed requirements and guidance.	This work was supported by Natural Science Foundation of China (81825019).
**Competing Interests**<br><br>On behalf of all authors, disclose any competing interests that could be perceived to bias this work.<br><br>This statement will be typeset if the manuscript is accepted for publication.<br><br>Review the instructions link below and PLOS NTDs' competing interests policy to determine what information must be disclosed at submission.	We declare no competing interests.
**Data Availability**<br><br>Provide a **Data Availability Statement** in the box below. This statement should detail where the data used in this	Data will be made available upon request made to the corresponding author.

submission can be accessed. This statement will be typeset if the manuscript is accepted for publication.<br><br>Before publication, authors are required to make all data underlying their findings fully available, without restriction. Review our PLOS Data Policy page for detailed information on this policy. Instructions for writing your Data Availability statement can be accessed via the Instructions link below.	

Powered by Editorial Manager® and ProduXion Manager® from Aries Systems Corporation

Click here to access/download;Manuscript;Manuscript.docx

Human infection with respiratory adenovirus in China: a systematic review and meta-analysis

Mei-Chen Liu[1,2¶], Qiang Xu[2¶], Ting-Ting Li[2,3], Tao Wang[2], Bao-Gui Jiang[2], Chen-Long Lv[2], Xiao-Ai Zhang[2], Wei Liu[1,2*], Li-Qun Fang[1,2,3*]

[1]School of Public Health, Anhui Medical University

[2]State Key Laboratory of Pathogen and Biosecurity, Beijing Institute of Microbiology and Epidemiology, Beijing, 100071, P. R. China

[3]School of Public Health, Guizhou Medical University

[*]Corresponding author

Li-Qun Fang or Wei Liu, State Key Laboratory of Pathogen and Biosecurity, Beijing Institute of Microbiology and Epidemiology, 20 Dong-Da Street, Fengtai District, Beijing 100071, P. R. China, fang_lq@163.com (LQF) or lwbime@163.com (WL).

[¶]These authors contributed equally to this work.

Abstract

Background Human adenovirus (HAdV) is a major pathogen that causes respiratory tract infections (ARTI) and frequently associated with outbreaks. The HAdV prevalence and the predominant types ~~that were~~ responsible for ~~the~~ ARTI outbreaks ~~remained~~ obscure in China.

Methods A systematic review was performed to retrieve literature that reported outbreaks or etiological surveillance of HAdV in China from January 2009 to March 2021. The genetic characteristics and epidemiological characteristics of HAdVs were investigated.

Results A total of 950 articles (91 about outbreaks and 859 about etiological surveillance) meeting the selection criteria were included. Predominant HAdV types from etiological surveillance studies differed from those in outbreak events. Among the 70 outbreaks for which the HAdVs were typed, nearly half (45·71%) were caused by HAdV-7 with an overall attack rate of 22·32% after meta-analysis. Military camp and school were predominant settings of outbreaks with HAdV-55 and HAdV-7 respectively identified as the leading type and with significantly different seasonal pattern and attack rate. Among 859 hospital-based surveillance studies, positive detection rates of HAdV-3 (32·73%) and HAdV-7 (27·48%) were significantly higher than other types. Clinical manifestations mainly depended on HAdV types and patient age with significant discrepancy among them, and infection with HAdV-55 tends to develop pneumonia with a poorer prognosis, especially for children <5 years old.

Conclusions This study improves the understanding of epidemiological and clinical features of HAdV infections and outbreaks with different types, and helps to inform future surveillance and control efforts in different settings.

Keywords: Human adenovirus; China; respiratory tract infections; Meta-analysis.

Introduction

Infection with HAdV causes a broad spectrum of clinical illnesses that varies depending on the infecting types: pharyngoconjunctival fever, keratoconjunctivitis, pneumonia, hemorrhagic cystitis, gastroenteritis, acute respiratory disease, severe disseminated disease, cardiomyopathy, and encephalitis[1]. Even unexplained liver injury or hepatitis were reported by two recent independent studies of UK children with HAdV- 2 infection, suggesting that HAdV-2 may trigger liver damage through the immune mechanisms of genetically predisposed children.[2, 3] There are at least 113 recognized HAdV types (http://hadvwg.gmu.edu/), which are assigned to seven subgroups (A–G) according to biophysical, biochemical, and genetic characteristics, with marked differences in tissue tropism and clinical manifestations[4]. The types most commonly found in respiratory samples belong to HAdV species C (HAdV-C1, HAdV-C2, HAdV-C5, and HAdV-C6) and to HAdV species B, subspecies B1 (HAdV-B3 and HAdV-B7) and B2 (HAdV-B14), which are endemic and epidemic respectively in pediatric populations[5].

In recent years, new serotypes or subspecies were increasingly recognized by using of phylogenetic analysis, which arise from genome recombination between the hexon gene, fiber, and penton genes. For any of the emerging new types or recombinant strains, high potential of spreading widely and even epidemic outbreaks might be resulted, due to the lack of herd immunity and specific vaccine intervention, posing severe threats to public health[6-9]. Acute respiratory infection caused by HAdV is the leading cause of morbidity in military forces worldwide. Since 1971, U.S. military recruits have been vaccinated with oral HAdV-4 and HAdV-7 vaccines, which has significantly decreased the epidemics of HAdV in the military[10],[11].

In recent years, there has been an increase in studies from hospital-based surveillance, reflecting a growing awareness of the importance of HAdV as respiratory pathogens. In general, there is still a lack of data on the HAdV prevalence

and the predominant type ~~that were~~ responsible for ~~the~~ ARTI sporadic outbreaks or epidemics in China.

Here we conduct a systematic review of all published research articles on outbreak investigation and active surveillance of HAdV at the nation-wide level, between January 2009 and March 2021 to evaluate the HAdV prevalence, types, seasonality, as well as to characterize patients' demographic and clinical data. This information might help to comprehensively understand the epidemic patterns of HAdV in China and support the adoption of targeted prevention and control measures.

Materials and Methods

This review was conducted according to the Preferred Reporting Items for Systematic Reviews and Meta-Analyses (PRISMA) statement, and has been registered with the international prospective register of systematic reviews (PROSPERO) (International Prospective Register of Ongoing Systematic Reviews) (CRD42022303015)[12].

Search strategy and selection criteria

Literature search was performed from the major databases including the PubMed database (https://pubmed.ncbi.nlm.nih.gov/), China National Knowledge Infrastructure (CNKI) (http://www.cnki.net/), Chongqing VIP (CQVIP) (http://www.cqvip.com) and Wanfang databases (http://www.wanfangdata.com.cn/), with the keywords ('HAdV' OR 'adenovirus' [Title/Abstract]) AND ('respiratory' [Title/Abstract] OR 'pneumonia' [Title/Abstract]), AND ('China' OR 'the mainland of China' OR 'Chinese mainland' OR 'Taiwan' OR 'Hong Kong' OR 'Macau' OR 'Macao' [Title/Abstract]) (Appendix1 p 2). All the articles published between January 2009 and March 2021 were searched without language limitations.

We included studies of human infection with HAdV, across all settings (i.e., hospital, community, long-term care) and among all age groups (pediatric and adult patients). We included etiological surveillance studies and outbreak investigation , but

excluded reviews, editorials, letters, case studies, randomized controlled trials and experimental studies. Studies were eligible if they explicitly described the total number of individuals tested and those that were positive for HAdV infections in humans. The following articles were excluded: (1) drug, vaccine trials, mechanism studies, animal experiments or reviews for HAdV; (2) etiological surveillance studies with sampling size <100 for laboratory test or HAdV positive detection <10; (3) describing cases imported from abroad after international travel; (4) lacking information about methods of laboratory diagnosis, specimens tested; (5) evaluations on laboratory methods for HAdV; (6) study period beyond the duration 2009–2020; (Appendix1 pp 3–4).

Titles and abstracts of the retrieved studies were screened using Endnote X9 independently by two reviewers (MCL and TTL) to identify studies potentially eligible for inclusion, and then the full texts were retrieved and independently assessed for eligibility. Discrepancies between reviewers were resolved by consensus or a third reviewer (QX). Studies potentially describing overlapping data were noted and the duplication were removed (e.g., same hospital and population during an overlapping time period).

Data extraction and variable definition

One of the authors (MCL) extracted data from included studies using a standardized data collection form. The following variables were collected: reference id, author, publish year, study sites, start and end dates, name(s) of healthcare facility; study design (surveillance study, outbreak investigation), outbreak setting (school/daycare outbreaks, healthcare comprised of hospitals and long-term care facilities, military camps, swimming pools), age group, patient population, mean or median age, gender proportion, laboratory test methods (molecular, serological) and type of HAdV, sample size, absolute number or rate of positive detection, presence of clinical symptoms or syndromes of patients if reported (Appendix1 pp 5–6). For

quality assurance, another two authors (QX, TW) randomly sampled 25% of recorded data to confirm accuracy and completeness.

For definition of outbreak event, all those recognized and reported outbreaks related to HAdV by health agencies were included. Otherwise, an outbreak event was defined as a number of clustered HAdV cases with a higher incidence than the average or expected incidence for a region where the cases occur. All the events had to be laboratory confirmed, e.g., etiological pathogen determined to be HAdV by molecular methods (PCR) or serological methods (ELISA, IFA), while those outbreaks reporting suspected HAdV without laboratory ~~confirm~~ for HAdV were not included in the analysis. For outbreak investigation, we extracted additional information regarding the exact date of outbreak, attack rate , numbers of primary cases, persons at risk, and number of secondary cases if available. For articles reporting more than one outbreak, data were separately extracted for each outbreak. For outbreaks reported in multiple publications, we included the one that reported more detailed data.

Four age groups were defined for comparison, including children (<5 years old), ~~adolescence~~ (5–17 years old), adult (18–59 years old), the elderly (≥60 years old). When a study did not mention any age information, the all-age groups were specified. Seven regions were defined according to the ecoclimatic characteristics, i.e., Northeast China, North China, Inner Mongolia-Xinjiang, Qinghai-Tibet, Southwest China, Central China, and South China[13].

Meta-analysis

We performed meta-analysis to evaluate demographic characteristics of patients, attack rate, or positive rate for HAdV. Briefly, the pooled proportion and 95% CI were estimated using the inverse variance combined with fixed effects or random effects models depending on the degree of the heterogeneity between studies. Heterogeneity was quantified using the statistic Higgin's I^2, when its value was greater than 50%, random effects model was used, otherwise, fixed effects model was

applied[14]. We performed meta-analysis on 105 articles with a clinical manifestations study size greater than 20 patients, to estimate the clinical manifestations that were related to different HAdV types. For those clinical manifestations which were mentioned in only one study, the proportion was calculated without a 95% CI estimated (Appendix2). All maps were produced using the ArcGIS 10.7 software. The Meta program package in R 4.1.2 software was used to merge the rates and draw forest plots. All analyses were conducted with R 4.1.2 software.

Results

Temporal and spatial features of publications and patients

A total of 5056 studies published from January 2009 to March 2021 were identified and after duplicate removal, 3874 studies underwent title and abstract screening, among ~~whom~~ 1329 were assessed via full-text screening. We included 950 studies (881 in Chinese and 69 in English) in the final analysis, comprised of 859 surveillance studies involving 119 838 patients and 91 outbreak investigations involving 15 940 patients (Fig 1, Appendix3).

Fig 1. ~~The flow~~ diagram of the literature review. Literature search was performed from the major databases including the PubMed database (https://pubmed.ncbi.nlm.nih.gov/), China National Knowledge Infrastructure (CNKI) (http://www.cnki.net/), Chongqing VIP (CQVIP) (http://www.cqvip.com) and Wanfang databases (http://www.wanfangdata.com.cn/), we included 950 studies (881 in Chinese and 69 in English) in the final analysis.

Of the 91 articles reporting 97 outbreak events, 68 (74·73%) were published ~~during~~ 2014—2019, with the highest number of articles published in 2014 (14 articles), followed by 2017 (13 articles) (Fig 2A). Of the 859 surveillance studies, 594

(69·15%) were published ~~during~~ 2015-2020, with the largest number published in 2015 (135 articles), followed by 2016 (103) (Fig 2C).

Fig 2. Number of recruited studies by ~~publish~~ year of HAdV in China, 2009–2021. (A) number of outbreaks articles group by ~~publish~~ year, (B) number of outbreak types group by ~~publish~~ year, (C) number of surveillance articles group by ~~publish~~ year, (D) number of surveillance types group by ~~publish~~ year. Other rarely seen types including HAdV-21 (with 30 patients report), HAdV-57 (18), HAdV-31 (five), HAdV-50 (two), HAdV-12 (one), HAdV-35 (one), and HAdV-104 (one).

A comparable number of outbreaks took place in Northern and Southern China (Table 1). The geographic discrepancy of seasonal timing was shown for the outbreaks, with most of the outbreak events occurring in the winter season in Northern China (28/46), while ~~showed~~ a dual seasonal timing in Southern China, ~~observed~~ at the turn of spring and summer and winter separately (44/51) (Fig 3A). The overall attack rate was estimated to be 15·91% (95% CI: 13·85–17·98). A higher attack rate was observed in Northern China than in Southern China (19·01%, 95% CI: 14·42–23·60 versus 13·53%, 95% CI: 11·28–15·77). When the outbreak settings were compared, the highest attack rate was observed in military camps (23·55%, 95% CI:18·02–29·07), followed by swimming pools (22·47%, 95% CI: 12·49–32·45), hospitals (19·75% , 95% CI: 8·64–30·86) and schools (6·19%, 95% CI: 4·92–7·46) (Fig 3B). In contrast with the outbreak events, surveillance study reported comparable interregional positive rate (4·13%, 95% CI: 3·95–4·31 in Southern China, 4·15% , 95% CI: 3·95–4·34 in Northern China), and the overall detection rate was estimated to be 4·21% (95% CI: 4·07–4·34) (Fig 3C, Appendix1 pp 7–11)

Table 1. Attack rate and positive detection rate by meta-analysis of HAdV by areas, season, age, settings and types.

	Outbreak events			Surveillance		
	Number of outbreak investigation articles (No. of outbreaks)	Cases	Attack rate by meta- analysis % (95% CI)*	Number of surveillance investigation articles	Cases	Detection rate by meta-analysis % (95% CI)
Number	91 (97)	15940	15·91 (13·85, 17·98)	859	119838	4·21(4·07, 4·34)
Mortality	4 (4)	4	-	9	37	-
Areas**						
Northern	44 (46)	9182	19·01 (14·42, 23·60)	283	19955	4·15 (3·95, 4·34)
Southern	48 (51)	6758	13·53 (11·28, 15·77)	584	97162	4·13 (3·95, 4·31)
Season						
Spring	19 (20)	899	5·92 (4·47, 7·36)	39	2047	5·21 (4·35, 6·07)
Summer	16 (17)	440	16·76 (12·47, 21·04)	39	1780	4·64 (3·82, 5·47)
Autumn	16 (17)	394	5·71 (3·41, 8·01)	36	1278	3·62 (2·95, 4·29)
Winter	40 (43)	8453	22·65 (16·33, 28·96)	37	2042	3·54 (2·83, 4·24)
Age						
Child	6 (6)	121	12·78 (7·95, 17·62)	174	10671	4·04 (3·76, 4·31)
Adolescence	40 (43)	2042	6·52 (5·5, 7·55)	8	531	4·45 (2·55, 6·36)
Adult	44 (47)	13734	23·56 (18·31, 28·8)	28	1219	3·44 (2·76, 4·12)
The elderly	0 (0)	0		9	229	2·81 (1·84, 3·78)
All-age groups	1 (1)	43	25·29	644	107188	4·29 (4·12, 4·45)
Settings						
School	41 (43)	3386	6·19 (4·92, 7·46)	0	0	
Military camp	37 (40)	11849	23·55 (18·02, 29·07)	0	0	
Hospital	5 (5)	142	19·75 (8·64, 30·86)	859	119838	4·21(4·07, 4·34)
Swimming pool	9 (9)	563	22·47 (12·49, 32·45)	0	0	

Types†						
HAdV-1	0 (0)	0	-	40	466	6·70 (5·39, 8·01)
HAdV-2	0 (0)	0	-	44	772	8·90 (7·31, 10·50)
HAdV-3	9 (11)	711	5·55 (2·63, 8·48)	60	2869	32·73 (22·13, 43·34)
HAdV-4	7 (7)	150	8·75 (0·00, 21·30)	30	137	2·07 (1·41, 2·74)
HAdV-5	0 (0)	0	-	38	249	3·55 (2·78, 4·32)
HAdV-6	0 (0)	0	-	21	83	1·97 (1·23, 2·70)
HAdV-7	30 (32)	7048	22·32 (14·78, 29·86)	59	2518	27·48 (17·04, 37·91)
HAdV-11	0 (0)	0	-	4	186	16·14 (1·89, 30·40)
HAdV-14	3 (4)	92	8·83 (6·31, 11·35)	12	43	2·01 (0·92, 3·09)
HAdV-21	0 (0)	0	-	7	30	0·87 (0·17, 1·57)
HAdV-31	0 (0)	0	-	4	5	0·33 (0·00, 0·73)
HAdV-55	16 (16)	4043	27·18 (19·16, 35·20)	25	258	4·70 (3·40 , 6·00)
HAdV-57	0 (0)	0	-	8	18	1·01 (0·51, 1·50)

* 53 outbreaks recording attack rate were included in the total; 2, 26, 24 and 1 outbreaks recording attack rate were included in the children age group, adolescence age group, adult age group and all age groups, respectively; 13, 8, 6 and 25 outbreaks recording attack rate were included in the spring, summer, autumn and winter group, respectively; 25 and 28 outbreaks recording attack rate were included in the north and south group, respectively; 22, 2, 6 and 23 outbreaks recording attack rate were included in the military, hospital swimming pool and school group, respectively; 4, 2, 17, 1 and 9 outbreaks recording attack rate were included in the HAdV-3, HAdV-4, HAdV-7, HAdV-14 and HAdV-55 group, respectively.

** Of the 848 articles mentioning locations, 19 mentioned both northern and southern China.

†HAdV type with case No> 5 were included, other rarely seen types ~~including~~ HAdV-50 (2), HAdV-12 (1), HAdV-35 (1), and HAdV-104 (1).

Fig 3. Attack rate of outbreak and detection rate of surveillance for HAdV in China. (A) attack rate by region, (B) attack rate by settings, (C) detection rate by regions, (D) attack rate by age groups.

(A, B, D) The bars indicate the number of outbreaks, and the intervals indicate the attack rate and 95% CI which is obtained by meta-analysis. (C) The bars indicate the number of surveillance cases, and the intervals indicate the detection rate and 95% CI which is obtained by meta-analysis.

Demographic characteristics of patients

The highest number of outbreak events were observed in the adult group (47), followed by adolescents (43) and children (6). The highest attack rate was in adults (23·56%, 95% CI: 18·31–28·80), followed by children (12·78%, 95% CI: 7·95–17·62) and adolescents (6·52%, 95% CI: 5·50–7·55). An age pattern of HAdV infection was shown from the surveillance data, with higher positive rat observed in children (4·04%, 95% CI: 3·76–4·31) and adolescents (4·45%, 95% CI: 2·55–6·36) than those of the two older groups (Table 1). Seasonal pattern differed among age groups. For children and adolescent, over half of the outbreaks occurred in autumn and spring (29 of 49 outbreaks), while for adult groups, most outbreaks occurred in winter (38/47) (Fig 3D, Appendix1 pp 12).

Among 97 outbreaks that reported settings, the highest number was reported in schools (44·33%, 43/97), followed by military camps (41·24%, 40/97), swimming pools (9·28%, 9/97), and hospitals (5·15%, 5/97). The seasonality was largely dependent on the outbreak settings, with most of the military camps outbreaks occurring in winter (85.00%, 34/40), and swimming pool outbreaks occurring in summer (88·89%, 8/9), while school outbreaks occurring in spring and autumn during school terms as expected (69·77%, 30/43). A total of 41 deaths with confirmed HAdV infection were reported from 13 articles, with an overall case fatality rate of 0.03% (Table 1).

Temporal and geographic pattern of HAdV types in China

Sequence information was available for 70 outbreak events involving 12 044 cases and 67 surveillance studies involving 7639 cases. The most common type responsible for outbreak events was HAdV-7, accounting for 45·71% (32/70) of the totally typed outbreaks, followed by HAdV-55 (16/70), HAdV-3 (11/70), HAdV-4 (7/70), and HAdV-14 (4/70) (Fig 2B). The case numbers involved in outbreaks caused by HAdV-7, HAdV-55 and HAdV-3 were 7048, 4043 and 711, respectively, based on which the attack rate of HAdV-55 was estimated to be 27·18% (95% CI: 19·16–35·20), which was significantly higher than that of HAdV-7 (22·32%, 95% CI: 14·78–29·86) and HAdV-3 (5.55%, 95% CI: 2.63–8.48) (Table1). A difference of the predominant HAdV type in outbreak events was shown between Northern China and Southern China. Among all the outbreaks reporting HAdV types and study sites, HAdV-55 (12 events involving 3841 cases) and HAdV-7 (17 events involving 2614 cases) were predominant in Northern China, while HAdV-7 (15 events involving 4434 cases), HAdV-3 (seven events involving 640 cases), and HAdV-55 (four events involving 202 cases) were predominant in Southern China (Fig 4A). According to the ecological regions of China, we found that HAdV-55 was ~~dominated~~ pathogen for outbreak events in four regions including Inner Mongolia-Xinjiang, North China, Qinghai-Tibet, and South China, while HAdV-7 was ~~dominated~~ in the others including Northeast China and Central China, expecting for one region without reporting outbreaks (Southwest China) (Appendix1 p15).

Fig 4. Comparison of types by region, age, and setting. (A) comparison of ~~the~~ types of outbreaks in different regions, (B) comparison of the types of surveillance in different regions, (C) comparison of the types of outbreaks in different age groups, (D) comparison of the types of outbreaks in different settings.

(A, C, D) Solid circles indicate the number of outbreaks, and hollow circles indicate the number of cases. (B) Solid circles indicate the number of surveillances, and hollow circles indicate the number of cases.

The predominant HAdV types from etiological surveillance studies differed from those in outbreak events. The most common type was HAdV-3 (2869 cases), followed by HAdV-7 (2518), HAdV-2 (772), HAdV-1 (466), HAdV-55 (258), and HAdV-5 (249) (Fig 2D). The positive rate of HAdV-3 was determined as 32·73% (95% CI: 22·13–43·34), which was significantly higher than that of HAdV-7 (27·48%), HAdV-11 (16·14%), HAdV-2 (8.90%), HAdV-1 (6.70%), HAdV-55 (4.70%), and. HAdV-5 (3.55%). Both HAdV-3 and HAdV-7 were the common types identified in northern and southern China (Fig 4B, Table1).

Among 70 outbreaks providing information on age and HAdV type, HAdV-7 (25) and HAdV-55 (15) were responsible for all the 40 outbreaks reported in the adult group; HAdV-7 (3) and HAdV-4 (3) were determined in the six outbreaks reported from children, while a higher diversity of types were observed in the 24 outbreaks reported in the adolescent group, including HAdV-3 (11), HAdV-4 (4), HAdV-7 (4), HAdV-14 (4), HAdV-55 (1) (Fig 4C). Among the 70 outbreaks with reported settings and types, HAdV-7 (21) and HAdV-55 (12) were responsible for all the 33 outbreaks in military camps, which involved 5111 and 3674 cases respectively. HAdV-55 (3 outbreaks) and HAdV-7 (2) were responsible for the five outbreaks in hospitals, with 113 and 29 cases involved respectively. HAdV-3 (2 outbreaks), HAdV-4 (1), and HAdV-7 (2) were determined to be responsible for the five swimming pool outbreaks, with 199, 147, and nine cases involved respectively. Five HAdV types were responsible for the 28 outbreaks in schools, main including HAdV-3 (9), HAdV-7 (7), HAdV-4 (6), HAdV-14 (4), and HAdV-55 (2), with 1761 cases with HAdV-7 and 512 cases with HAdV-3 infection taking the large part of the case (Fig 4D).

Clinical manifestations

Altogether 105 articles with HAdV cases greater than 20 were included for the investigation of clinical manifestations. Among children with HAdV infection, cough was the most prevalent, identified in 76·47% cases, followed by pneumonia (66·56%), expectoration (56·28%), respiratory failure (11·05%), breathing difficulties (34·69%), running nose (29·58%), vomiting (25·00%), and diarrhea (16·87%). Among adolescent ~~group,~~ tonsillar enlargement (70·89%), pneumonia (21·69%) and vomiting (15·43%) were frequently seen. Among adult ~~group,~~ less diverse clinical presentations were seen, including expectoration (31·19%), running nose (8·05%) and vomiting (4·60%) (Fig 5A, Appendix1 pp 13).

Fig 5. Clinical manifestations of HAdV infection by age groups and types.
(A,B)The bars indicate the number of articles, and the intervals indicate the detection rate and 95% CI which is obtained by meta-analysis.

Comparison among HAdV types revealed HAdV-3 infection was related to higher frequency of cough (79·91%), running nose (20·59%) and vomiting (20·59%), compared with HAdV-55 and HAdV-7. HAdV-55 was related to higher frequency of tonsillar enlargement (96·91%), pneumonia (27·91%), while lower presence of cough (56·61%), expectoration (45·10%), running nose (6·90%), and vomiting (3·37%), diarrhea (1·50%), compared with HAdV-3 and HAdV-7. HAdV-7 was related to higher presence of expectoration (60·59%), breathing difficulties (26·80%), compared with HAdV-3 and HAdV-55 (Fig 5B, Appendix1 pp 14).

Discussion

The current study provided a most comprehensive and up to date estimation on the attack rate/positive rate of HAdV as well as the dominant genogroups, that differed across age, setting and seasons. Attack rates were significantly higher in

outbreaks related to military camps and in the winter season. HAdV55, HAdV7 and HAdV3 were the major causative agents for the outbreak setting, however, outbreaks in military camps were more likely to be associated with HAdV7 and HAdV55, while outbreaks in other setting were associated with more HAdV genotypes, particularly for the school outbreaks. This was also in line with the higher diversity of types in the adolescent group, who constituted the major part of school outbreaks. These genotyping results contrasted with the surveillance study where HAdV-3 and HadV-7 were most frequently determined when an all-age group was studied. The current surveillance finding was highly consistent with the prior epidemiological investigation performed in China, where HAdV-3 and HAdV-B7 were most frequently detected among acute respiratory distress syndrome patients[15-18].

We found that school-related outbreaks were associated with the lowest attack rate, suggesting either a less confined space that limited the transmission or the lower transmission capacity relate to different HAdV types. We also revealed a different situation from that shown in other countries, for example, in the American troops, HAdV-4, HAdV-B7 and HAdV-B14 act as the predominant strains sequentially isolated from the outbreaks[7, 19-21]. HAdV vaccination program against these two-outbreak related genotypes in this high-risk population is urgently needed. Our meta-analysis showed that overall attack rate of adenovirus infection in outbreaks was estimated ~~by~~ 15·91% (95% CI: 13·85–17·98), which is comparable with the outbreak among college students in Pennsylvania, USA, of which a 15% (44/288) attack rate was reported based on the test of nasopharyngeal swabs for HAdV[22].

Our study for the first time revealed ~~an obvious~~ seasonal pattern for the outbreak events. The school outbreaks occurred mostly at the beginning of new school years, while the military outbreaks occurred at the recruit training seasons. A Korean study from 2013 to 2018 showed that HAdV was the most frequently detected respiratory virus in military recruits (6646/1 4630, 45·4%)[23]. HAdV has been implicated in

over half of the febrile respiratory illness cases reported at recruit training center clinics.

A total of 17 respiratory adenovirus infection types have been reported in China, mainly HAdV-3, HAdV-7 and HAdV-55. Outbreaks were dominated by HAdV-7 and HAdV-55, while other types included type HAdV-3, HAdV-4 and HAdV-14. The main types of outbreaks in different settings differ, with school outbreaks having more types, including HAdV-7, HAdV-55, HAdV-3, HAdV-4 and HAdV-14, with HAdV-3 and HAdV-7 predominating; swimming pool outbreaks having mainly HAdV-3, HAdV-7 and HAdV-4, mainly in summer; hospital outbreaks and military outbreaks having mainly HAdV-7 and HAdV-55. The types of outbreaks vary by age group, with children predominantly HAdV-7, HAdV-4, adults predominantly HAdV-7, HAdV-55, and adolescents with more types of outbreaks, including HAdV-7, HAdV-55, HAdV-3, HAdV-4, and HAdV-14. There are more types detected in adenovirus surveillance, including HAdV-3, HAdV-7, HAdV-2, HAdV-1, HAdV-55, HAdV-5, etc. The most common types are HAdV-3 and HAdV-7 and there is an increasing trend ~~year on year~~, which may be related to the increasing number of infections and rising levels of surveillance. Differences in the main types in outbreak investigations and surveillance reports suggest that HAdV-55 is more infectious and more likely to lead to outbreaks, whereas HAdV-3 is less infectious than HAdV-55 and HAdV-7 and is easily detected during hospital surveillance. This may be because HAdV-55 is more symptomatic, more contagious and more likely to be reported. A study has shown that HAdV-7 replicates more robustly than HAdV-3, and promotes an exacerbated cytokine response, causing a more severe airway inflammation[24].

Overall, adenovirus circulates throughout the year, with slightly higher numbers reported during the summer and winter. Previous studies have shown that HAdV detection rates are positively correlated with the monthly mean temperature and sunshine duration, and negatively correlated with wind speed.[25] In our study, the main prevalent adenovirus types in China were HAdV-3, HAdV-7 and HAdV-55,

with other more frequent types such as HAdV-2, HAdV-4, HAdV-1 and HAdV-5. The main outbreak sites were schools and the military, with higher detection rates in the south than in the north. The prevalent typing varies between countries and regions, and a surveillance report in the United States from 2003–2016 showed that the common typing in the United States was HAdV-3, HAdV-2, HAdV-1, and HAdV-4[26]. An epidemiologic study based on HAdV molecular typing was conducted in the Korean military from January 2013 to April 2014, and HAdV-55 (42·0%) was the most frequently identified strain, followed by HAdV-4 (13·0%), HAdV-5 (1·4%), and HAdV-6 (1·4%)[27]. HAdV-55 is a relatively recently identified pathogen, which evolved from recombination between adenovirus 11 and 14[28, 29]. It was initially described as serotype 11a and was later re-labeled as HAdV-55 because of its recombinant genome[29]. HAdV-55 is mainly found in China and Korea[26].

The clinical presentation varies by age group and by type. Most adenovirus respiratory infections are light to moderate and self-limited; however, sometimes they may cause life-threatening conditions, comorbidities, and serious sequelae. The rate of pneumonia is higher in the children than in other age groups and, in addition, the children is more likely to have breathing difficulties. Attention should be paid to the occurrence of adenovirus pneumonia in children. The main symptoms are fever and tonsil enlargement in adolescents while the main symptoms are cough and tonsil enlargement in adults. Adolescents and adults have a stronger immune system and the symptoms are mainly mild. Compared to other types, HAdV-55 is more likely to cause pneumonia and has a high prevalence in the military camps, so attention should be paid to the prevention and control of adenovirus in the military camps.

There were two main limitations to this study. First, inherent to systematic reviews, our study was influenced by publication bias. Most outbreaks are reported by passive surveillance which may not be as comprehensive as in active surveillance, and also subject to reporting biases. Secondly, as our data came from different studies, and did not have the same variables. This may have increased the likelihood of

misclassification bias, also limited the number of variables that can be used for analysis.

Despite of these limitations, we have disclosed the incidence of HAdV and the major genotypes that differed over time, by location, and by characteristics such as patient age. Comparing and contrasting the features across diverse settings can help to attain an enhanced epidemiological and clinical understanding of human infections with different HAdV types, and thus enhancing the accuracy of HAdV surveillance systems.

Author Contributors

The author contributions are as follows. LQF and WL conceived, designed, and supervised the study. MCL, QX, TTL, TW, BGJ and CLL searched, screened, and assessed the publications. MCL and TTL created data extraction forms and extracted and analyzed the data. QX, TW, BGJ, CLL and XAZ helped with checking data and constructed the figures. QX, TW, BGJ and CLL provided statistical and clinical expertise in data analysis. MCL and QX wrote the drafts of the manuscript. MCL and QX interpreted the findings. LQF and WL commented on and revised drafts of the manuscript. All authors read the manuscript, provided feedback, and approved the final version.

Data Availability Statement

Data will be made available upon request made to the corresponding author.

Declaration of interests

We declare no competing interests.

Funding

This work was supported by Natural Science Foundation of China (81825019).

Acknowledgments

The authors would like to thank all patients who contributed data and clinicians who collected the data. This work was supported by Natural Science Foundation of China (81825019). The funders had no role in the design and conduct of the study; collection, management, analysis, and interpretation of the data; preparation, review, or approval of the manuscript; and decision to submit the manuscript for publication.

Reference

1. Lynch JP 3rd, Fishbein M, Echavarria M. Adenovirus. Semin Respir Crit Care Med. 2011;32:494-511.
2. Antonia Ho, Richard Orton, Rachel Tayler, et al. Adeno-associated virus 2 infection in children with non-A-E hepatitis. medRxiv, 2022.07.19.22277425.
3. Morfopoulou, S. et al. Genomic investigations of acute hepatitis of unknown aetiology in chilfren. medRxiv, 2022.07.28.22277963.
4. Seto D, Chodosh J, Brister JR, Jones MS. Members of the Adenovirus Research Community. Using the whole-genome sequence to characterize and name human adenoviruses. J Virol. 2011;85:5701-5702.
5. Mennechet FJD, Paris O, Ouoba AR, et al. A review of 65 years of human adenovirus seroprevalence. Expert Rev Vaccines. 2019;18:597-613.
6. Kajon AE, Lu X, Erdman DD, et al. Molecular epidemiology and brief history of emerging adenovirus 14-associated respiratory disease in the United States. J Infect Dis. 2010;202:93-103.
7. Ryan MA, Gray GC, Smith B, McKeehan JA, Hawksworth AW, Malasig MD. Large epidemic of respiratory illness due to adenovirus types 7 and 3 in healthy young adults. Clin Infect Dis. 2002;34:577-582.

8. Lu G, Peng X, Li R, et al. An outbreak of acute respiratory infection at a training base in Beijing, China due to human adenovirus type B55. BMC Infect Dis. 2020;20:537.
9. Mi Z, Butt AM, An X, et al. Genomic analysis of HAdV-B14 isolate from the outbreak of febrile respiratory infection in China. Genomics. 2013;102:448-455.
10. Lynch JP 3rd, Fishbein M, Echavarria M. Adenovirus. Semin Respir Crit Care Med. 2011;32:494-511.
11. Hoke CH Jr, Snyder CE Jr. History of the restoration of adenovirus type 4 and type 7 vaccine, live oral (Adenovirus Vaccine) in the context of the Department of Defense acquisition system. Vaccine. 2013;31:1623-1632.
12. Page MJ, McKenzie JE, Bossuyt PM, et al. The PRISMA 2020 statement: an updated guideline for reporting systematic reviews. BMJ. 2021;372:n71.
13. Wang LP, Zhou SX, Wang X, et al. Etiological, epidemiological, and clinical features of acute diarrhea in China. Nat Commun. 2021;12:2464.
14. Rowe WP, Huebner RJ, Gilmore LK, Parrott RH, Ward TG. Isolation of a cytopathogenic agent from human adenoids undergoing spontaneous degeneration in tissue culture. Proc Soc Exp Biol Med. 1953;84:570-573.
15. Deng J, Qian Y, Zhao LQ, Zhu RN, Sun Y, Tian R. [Identification and typing of adenovirus from acute respiratory infections in pediatric patients in Beijing from 2003 to 2012]. Chinese journal of virology. 2013;29:615-20.
16. Jin Y, Zhang RF, Xie ZP, et al. Prevalence of adenovirus in children with acute respiratory tract infection in Lanzhou, China. Virol J. 2013;10:271.
17. Lu QB, Tong YG, Wo Y, et al. Epidemiology of human adenovirus and molecular characterization of human adenovirus 55 in China, 2009-2012. Influenza Other Respir Viruses. 2014;8:302-308.
18. Cao B, Huang GH, Pu ZH, et al. Emergence of community-acquired adenovirus type 55 as a cause of community-onset pneumonia. Chest. 2014;145:79-86.

19. Binder AM, Biggs HM, Haynes AK, et al. Human Adenovirus Surveillance - United States, 2003-2016. MMWR Morb Mortal Wkly Rep. 2017;66:1039-1042.
20. Kajon AE, Lamson DM, St George K. Emergence and re-emergence of respiratory adenoviruses in the United States. Curr Opin Virol. 2019;34:63-69.
21. Kolavic-Gray SA, Binn LN, Sanchez JL, et al. Large epidemic of adenovirus type 4 infection among military trainees: epidemiological, clinical, and laboratory studies. Clin Infect Dis. 2002;35:808-818.
22. Biggs HM, Lu X, Dettinger L, Sakthivel S, et al. Adenovirus-Associated Influenza-Like Illness among College Students, Pennsylvania, USA. Emerg Infect Dis. 2018;24:2117-2119.
23. Ko JH, Woo HT, Oh HS, et al. Ongoing outbreak of human adenovirus-associated acute respiratory illness in the Republic of Korea military, 2013 to 2018. Korean J Intern Med. 2021;36:205-213.
24. Fu Y, Tang Z, Ye Z, et al. Human adenovirus type 7 infection causes a more severe disease than type 3. BMC Infect Dis. 2019;19:36.
25. Tian JY, Hu AH, Pan JG, et al. An outbreak of acute respiratory tract infection caused by adenovirus serotype 7 in a military camp in Shanxi province. J Pre Med Chin PLA. 2014;32:203–205.
26. Hang J, Kajon AE, Graf PCF, et al. Human Adenovirus Type 55 Distribution, Regional Persistence, and Genetic Variability. Emerg Infect Dis 2020;26:1497-1505.
27. Heo JY, Noh JY, Jeong HW, et al. Molecular Epidemiology of Human Adenovirus-Associated Febrile Respiratory Illness in Soldiers, South Korea. Emerg Infect Dis 2018;24:1221-1227.
28. Walsh MP, Seto J, Jones MS, et al. Computational analysis identifies human adenovirus type 55 as a re-emergent acute respiratory disease pathogen. J Clin Microbiol 2010;48:991-993.
29. Yang Z, Zhu Z, Tang L, et al. Genomic analyses of recombinant adenovirus type 11a in China. J Clin Microbiol 2009;47:3082-3090.

Figure 1

Click here to access/download;Figure;Figure 1.pdf

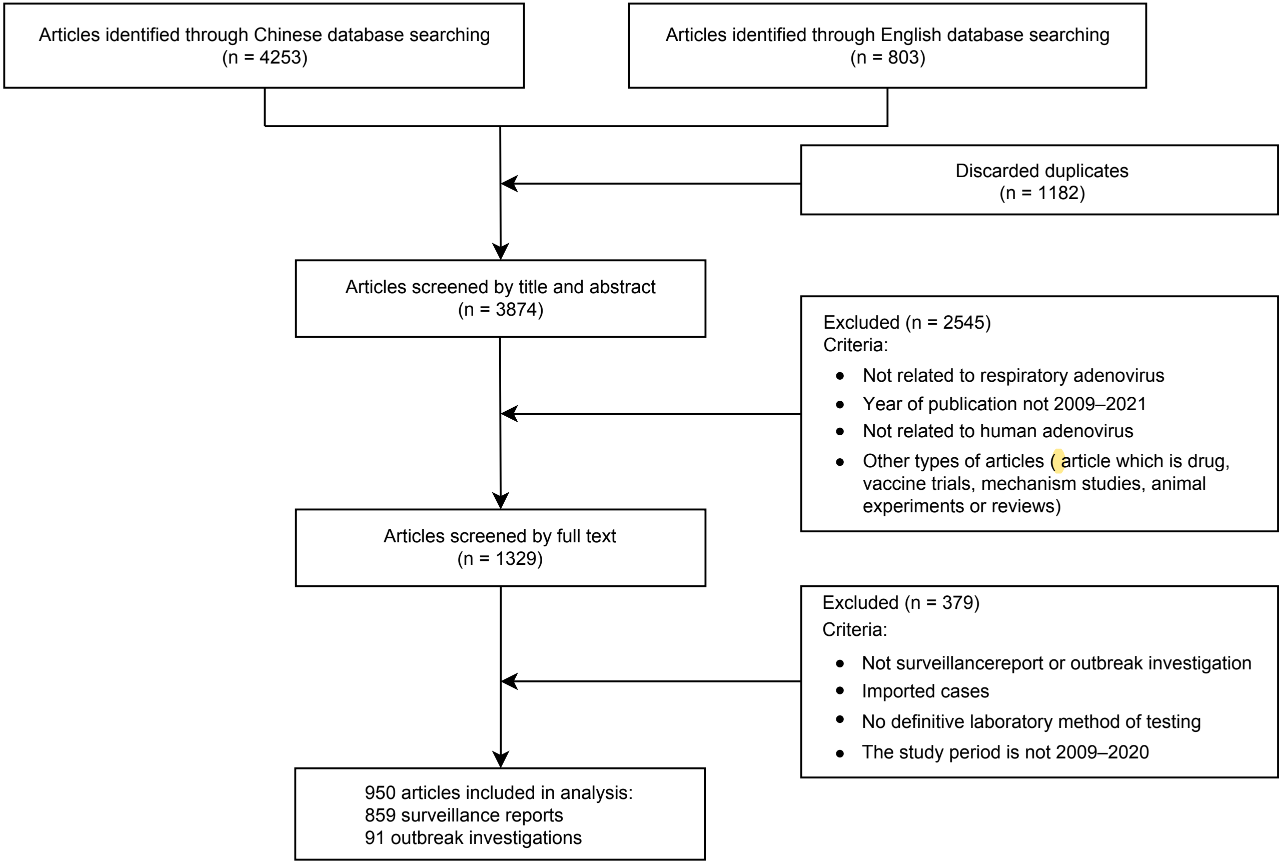

Figure 2

Click here to access/download;Figure;Figure 2.pdf

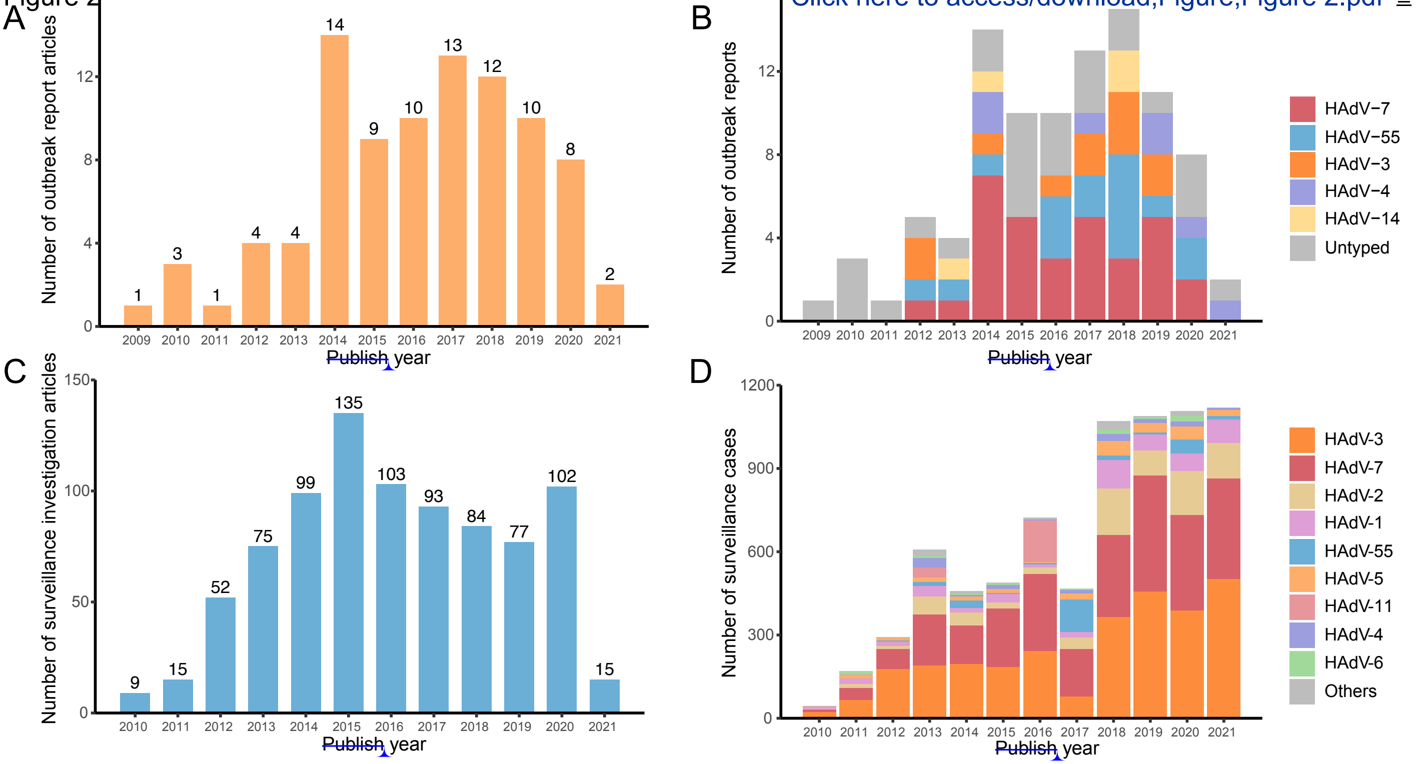

Figure 3

Click here to access/download;Figure;Figure 3.pdf

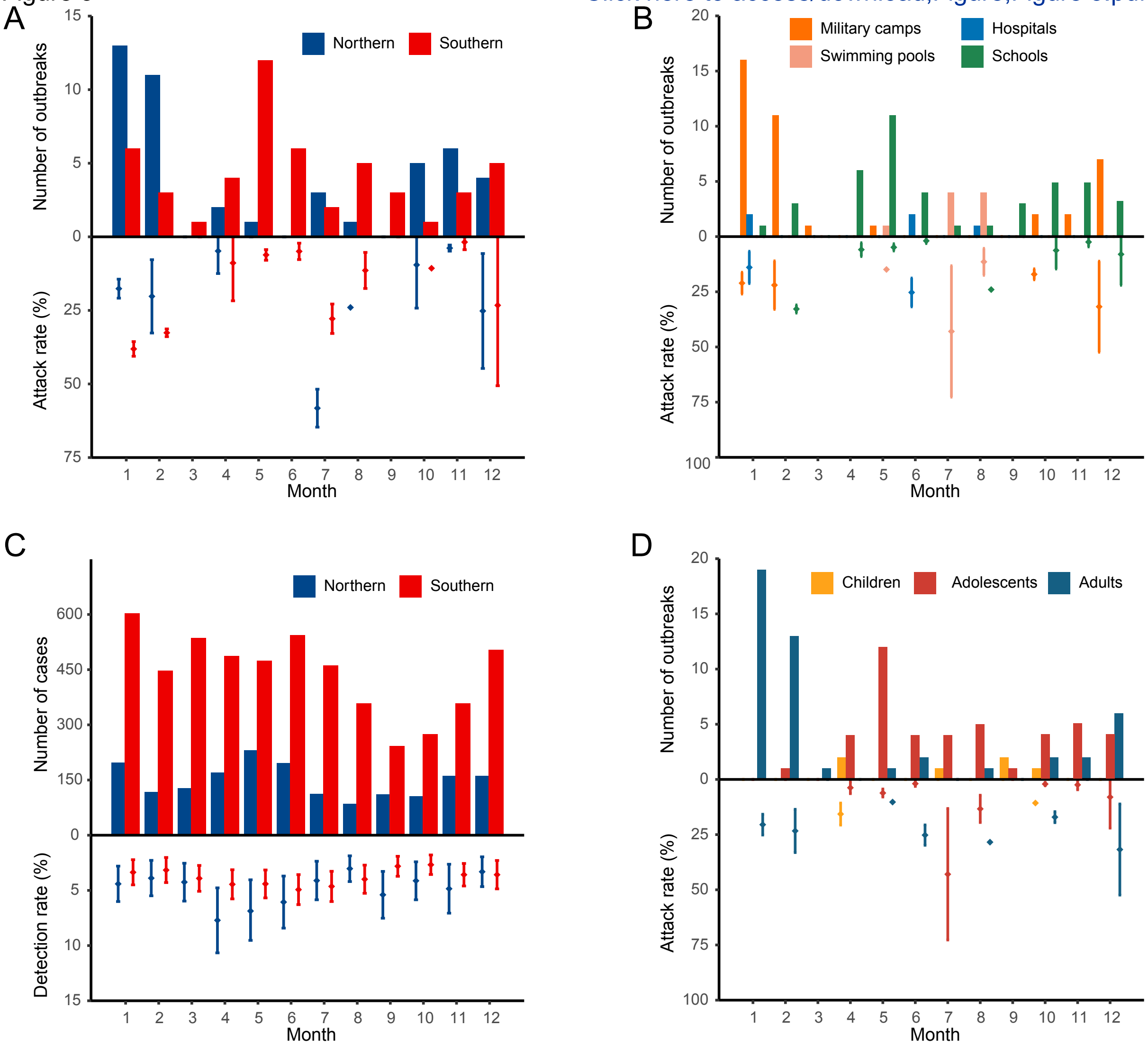

Figure 4

Click here to access/download;Figure;Figure 4.pdf

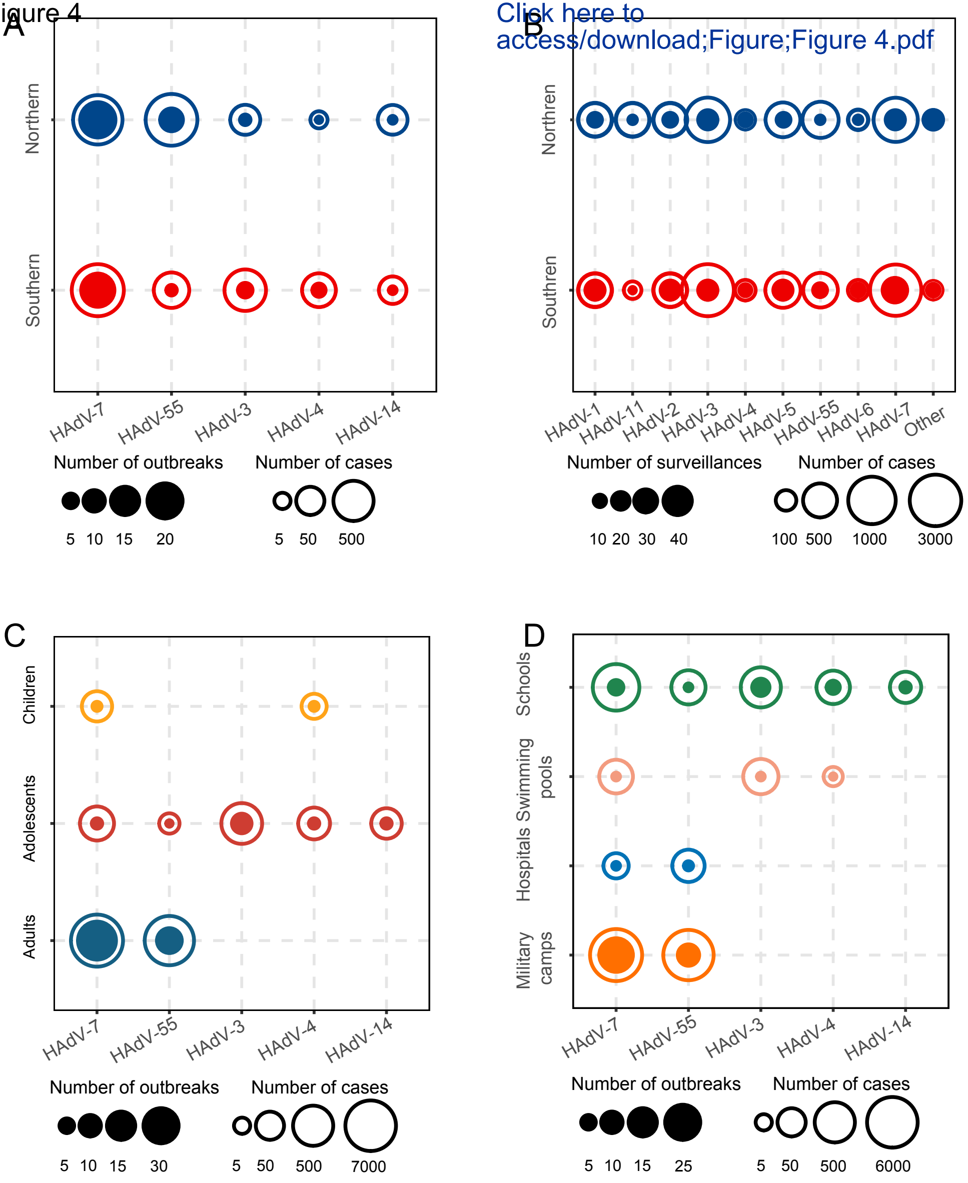

Figure 5

Click here to access/download;Figure;Figure 5.pdf

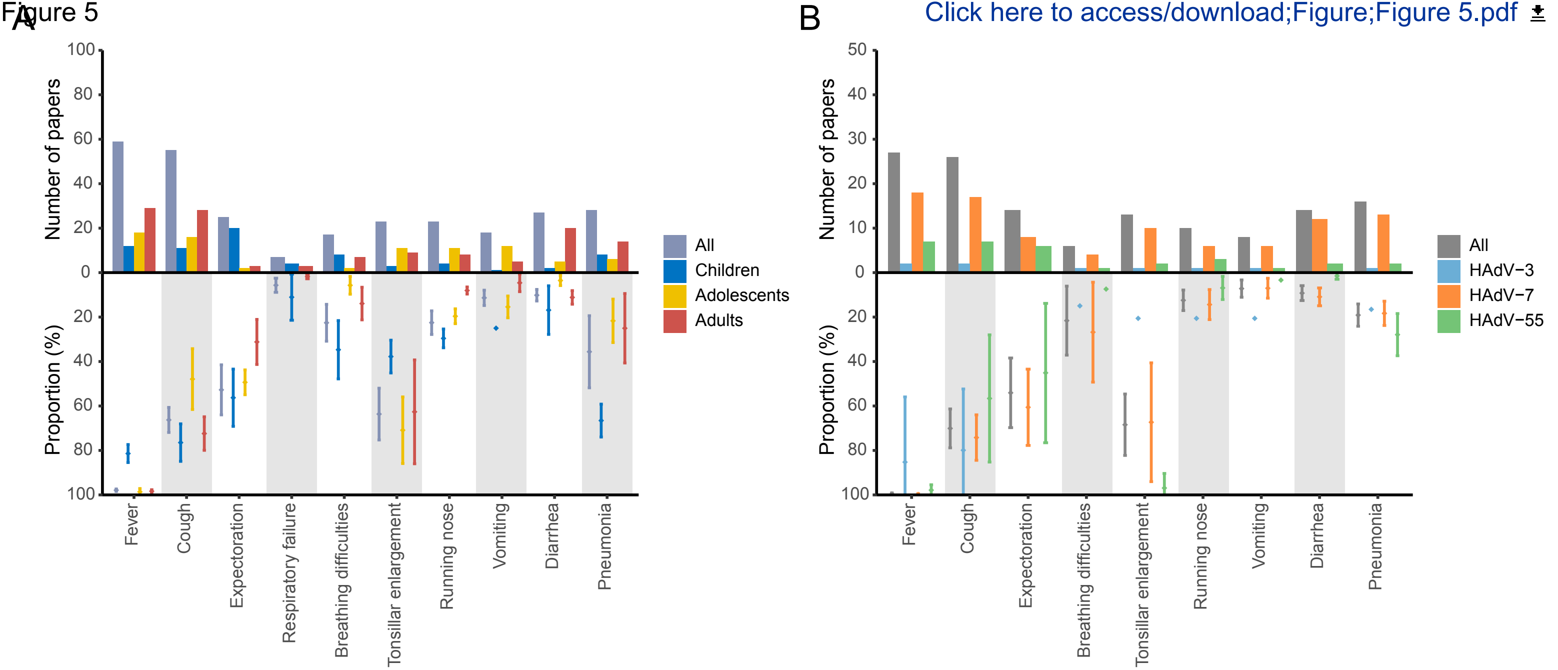

Click here to access/download
Supporting Information
Appendix1.pdf

Click here to access/download
Supporting Information
Appendix2.pdf

Click here to access/download
Supporting Information
Appendix3.pdf
