# Supplementary material for: Prevalence of human infection with respiratory adenovirus in China: A systematic review and meta-analysis
Source: PLoS Negl Trop Dis. 2023 Feb 22;17(2):e0011151. doi: 10.1371/journal.pntd.0011151 (PMC9987798; doi:10.1371/journal.pntd.0011151)
Supplement: Attachment — Submitted filename: Response Letter.docx [file pntd.0011151.s006.docx]

**Responses to the reviewers' comments**

**Title**

Reviewer #1:

It would be good to clarify if this article is a prevalence study or not.

**[Response] We appreciate the reviewer’s helpful suggestion. We have added the prevalence study to the title as: “Prevalence of human infection with respiratory adenovirus in China: a systematic review and meta-analysis”. (Page 1, Lines 1–2)**

**Abstract**

Reviewer #1:

Methods should be written in more detail about the study design, sampling, and clinical manifestation.

**[Response] Thanks for the reviewer’s suggestions. We have supplemented the study design, sampling, and clinical manifestation in the Abstract as: “****A systematic review was performed to retrieve literature that reported outbreaks or etiological surveillance of HAdV among ARTI patients in China** **from 2009 to 2020. Patient information was extracted from the literature to explore the epidemiological characteristics and clinical manifestations of the infection of various HAdV types.** **The study is registered with PROSPERO, CRD42022303015”. (****Page 2, Lines 24–28)**

**Introduction**

Reviewer #1:

1.The introduction needs to be improved by adding some information about the global prevalence, mortality, morbidity, and burden of HAdV both worldwide and in China.

**[Response] Many thanks for the reviewer’s helpful suggestions. We have supplemented the information about the global prevalence, mortality, morbidity, and burden of HAdV both worldwide and in China as: “A global study concluded that adenovirus infections accounted for 5–10% of respiratory infections in children and 1–7% in adults, and caused pneumonia in up to 20% of newborns and infants. In patients with severe HAdV pneumonia, the mortality rate may exceed 50%[12]. The positive detection rate of adenovirus was 3.9% and the mortality rate was 3% from 2004 to 2018 among inpatients hospitalized due to severe acute respiratory infection[13]. Studies in mainland China have shown that positive detection rate of adenovirus among ARTI patients was approximately 5.8%–13%, and the main affected groups were children and young adults[14]. Studies showed that the positive detection rate of adenovirus was 5.64% among hospitalized children with ARTI in Beijing from 2017 to 2018 and 6.9% in Zhejiang from 2018 to 2019[15,16]”. (Page 6, Lines 129–139)**

2.Page 4, line 75: “Here we conduct a systematic review of all published research...” isn't that a systematic review and meta-analysis?

**[Response]** **Thanks. We have corrected this sentence as “Here we conduct a systematic review and meta-analysis of all published research articles on outbreak investigation and etiological surveillance of HAdV among ARTI patients in China at the nation-wide level from January 2009 to 2020, to evaluate the HAdV prevalence, types, seasonality, as well as to characterize patients’ demographic and clinical data”. (Page 6, Line 1****42–156)**

**Methods**

Reviewer #1:

1.Page 5, line 109: Part 6 of the exclusion criteria says “Study period beyond the duration 2009-2020” while the authors indicated data extraction was conducted between Jan 2009 and Mar 2021. Also, in Appendix1 study period is 2009-2021 but in table S3 it is from 2009 to 2020. Please explain the difference.

**[Response] Thanks for the reviewer’s helpful comments. We extracted the literature in March 2021 for the study period from 2009 to 2020. We have addressed this issue throughout the manuscript.**

2.In appendix1 figure S1, I can't see the distribution of adenovirus typing pie chart for southwest China on the map!

**[Response]** **Many thanks for the reviewer’s helpful comments. Few studies reported the status of respiratory adenovirus infections in Southwest China, where** **no data has been reported on the** **adenovirus typing. We have supplemented the information in the notes of fig 1 in S2 File.**

3.There is not enough information about how the quality assessment was done! Please state the exact method that has been used, provide a complete checklist of risk of bias as an appendix, and address the references.

**[Response]** **We appreciate the reviewer’s valuable suggestions. Our exclusion criteria partially accounted for the quality of studies, e.g., The study requires an adequate sample size, complete outcome data, complete laboratory testing standards, specific testing criteria. To evaluate the methodological quality of the studies, two reviewers (MCL and TTL) independently assessed each study potentially eligible for inclusion. Discrepancies between reviewers were resolved by consensus or a third reviewer (QX). One of the authors (MCL) extracted data from included studies using a standardized data collection form. For quality assurance, another two authors (QX, TW) randomly sampled 25% of recorded data to confirm accuracy and completeness. We followed the PRISMA checklist to complete the meta-analysis. S4 File contains all included literature, and the full checklist has been provided in S1 File.**

4.Page 6, lines 131-133: “an outbreak event was defined...” is there any reference for this definition?

**[Response] Thanks for the reviewer’s helpful comments. We have supplemented a reference (Ref. 18) for this definition.**

5.Please report sensitivity and specificity of laboratory determination methods that have been used.

**[Response] Thanks for the reviewer’s valuable suggestion. It is pity that the information about sensitivity and specificity of laboratory determination methods is not available due to the data were from the literature, of which the related information was not reported.**

Reviewer #2:

The hypothesis and objectives of the work are clearly stated.

The selection of the articles is carried out properly.

Statistical analyzes are adequate.

**[Response] Many thanks for the reviewer’s positive comments.**

Reviewer #3:

The study is a systematic review and meta-analysis about human adenovirus (HAdV) infection associated with respiratory disease in China. The methodology is in accordance with a systematic review and met analysis article.

**[Response] Many thanks for the reviewer’s positive comments.**

**Results**

Reviewer #1:

There could have been data from HAdV vaccination in China among all age groups and a proper comparison of HAdV incidence between vaccinated and non-vaccinated individuals.

**[Response] Many thanks for the reviewer’s helpful suggestion. However, there is no available adenovirus vaccine in China up to now, and all patients in this study were considered not to be vaccinated.**

There could have been information about the most common type of HAdV separated by year from fig2B and fig2C.

**[Response] Many thanks for the reviewer’s helpful suggestions. We have supplemented a detailed description about the most common type of HAdV as: “****The most common type responsible for the outbreak events was HAdV-7, accounting for 45.71% (32/70) of all outbreaks with information about HAdV typing, followed by HAdV-55 (16/70), HAdV-3 (11/70), HAdV-4 (7/70), and HAdV-14 (4/70). HAdV-7 had the highest number of reported outbreaks in 2014, 2015 and 2017, while HAdV-55 (5) had the highest proportion of reported outbreaks in 2018 (Fig 2B).” (Page 17, Lines 473–478) “****Both HAdV-3 and HAdV-7 had been shown an increasing pattern in the number of reported cases during the study period (Fig 2D)”. (Page 19, Lines 527–529)**

Reviewer #2:

The results section describes very well the analysis carried out considering three different age ranges, and important to describe.

The graphs are well designed and display the results in an easy to view manner.

The comparative table is correct and presents valuable information comparing outbreaks and surveillance.

**[Response] Many thanks the reviewer’s positive comments.**

Reviewer #3:

A total of 5056 studies were identified, of which 950 articles met the inclusion criteria and were analyzed in the study. The study analyzed both articles from outbreak studies as well as from surveillance studies.

Presentations of results could be improved, especially concerning use of expressions and words not well explained in the text, grammar errors that make the text hard to understand, including in Figure legends.

**[Response] Many thanks for the reviewer’s helpful suggestions. We have a senior author with fluent English check the language throughout the manuscript carefully, and the presentations of results and figure legends have been improved in the revised manuscript.**

**Discussion**

Reviewer #1:

Page 17 line 3: “The prevalent typing varies...” presented common HAdV types in the United States from 2003-2016. It would be better if authors used the same time period for comparison as the current study. And please mention the most common types globally not just one country like the USA.

**[Response] We appreciate the reviewer’s helpful suggestion.** **We have added a corresponding description in the discussion as: “****The prevalent virus typing varies between countries and regions.** **Species B, C and D are the most common adenoviruses worldwide, of which species B and C could cause respiratory infections. For species B, a low prevalence of HAdV-7, HAdV-11 and HAdV-35 was reported globally, however, it is high prevalence for HAdV-35 in some counties from Africa with a positive detection rate of about 20% among HIV-infected patients, and a high prevalence of HAdV-7 was reported in China, United States and Belgium. For species C, HAdV-5 was the widely distributed species, and HAdV-2 usually had a high positive detection rate among healthy individuals and HIV-infected patients in China and developed countries[5]”. (Page 24, Line 690–704)**

**Reference**

Reviewer #1:

Please add journal issue after volume number.

**[Response] Thanks. Done as suggested.**

Reference order could have been reported by year, but it looks fine this way.

**[Response] Thanks. We have revised the order of references according to the year of publication (Data in S4 File).**

**Minor issues**

Reviewer #1:

There is no line number after page 10.

**[Response] Thanks. Done as suggested.**

Page 11, first paragraph line 5: rat ◊ rate

**[Response] Many thanks for the reviewer’s correction. We have made this correction as suggested.**

Reviewer #2: The discussion is correct, with updated bibliography.

The results support the discussion and the conclusion reached by the authors.

The limitations of the study are clearly stated in the discussion.

**[Response] Thanks for the reviewer’s positive comments.**

Reviewer #3: The study discusses the analyses performed and its findings, comparing with studies from some other countries. I suggest including a discussion about mortality associated with HAdV infection, comparing findings of the study (0.03 mortality) with other studies, for example the study by Pscheidt et al. (2020 - https://doi.org/10.1002/rmv.2189) that found 3% fatalities among patients hospitalized with severe respiratory infection and who were HAdV-positive.

**[Response] Many thanks for the reviewer’s helpful suggestions. We have supplemented the discussion about the** **mortality associated with HAdV infection, comparing findings of our study with other studies as: “****In our study, the mortality rate of adenovirus infection in patients with respiratory infections was about 0.03%, while a study in Rio Grande Do Sul, Brazil showed a higher mortality rate (3%) among hospitalized patients with severe acute respiratory infection****, which might be caused by a lower proportion of patients with severe respiratory infection in our study[13].” (Page 24, Line 678–682)**

**Summary and General Comments**

Reviewer #1: The manuscript “Human infection with respiratory adenovirus in China: a systematic review and meta-analysis” is a review of 950 articles that describes Human adenovirus (HAdV) prevalence, predominant types, clinical manifestation among different age groups and settings, national wide. The objective is interesting, although it needs some revisions before acceptance.

**[Response] We appreciate the reviewer’s positive comments and helpful suggestions.**

Reviewer #2: The manuscript entitled “Human infection with respiratory adenovirus in China: a systematic review and meta-analysis”, authored by Mei-Chen Liu et al, described the epidemiological and clinical features of HAdV infections in China, from January 2009 to March 2021. In addition, the genetic and epidemiological characteristics of HAdVs were investigated.

I want to emphasize that the work is very well written and concisely covers all the epidemiological characteristics of HAdV respiratory infections.

The work carried out is a very important contribution to the knowledge of the classical and molecular epidemiology of HAdV in China and also provides valuable information worldwide.

**[Response] Many thanks for the reviewer’s positive comments.**

Reviewer #3: The study is interesting and is worth publication, however thorough English revision is necessary. I recommend revision by a native English speaker.

Main comments and corrections were made in the pdf file, attached.

**[Response] Many thanks for the reviewer’s positive comments and corrections. We have a senior author with fluent English check the language carefully.**
