# Supplementary material for: Prevalence of human infection with respiratory adenovirus in China: A systematic review and meta-analysis
Source: PLoS Negl Trop Dis. 2023 Feb 22;17(2):e0011151. doi: 10.1371/journal.pntd.0011151 (PMC9987798; doi:10.1371/journal.pntd.0011151)
Supplement: Attachment — Submitted filename: Response letter.docx [file pntd.0011151.s008.docx]

**Responses to the reviewers' comments**

**Methods**

Reviewer #2: The objectives and design of the study are clear.

The statistical analysis performed was adequate.

**[Response] Many thanks for the reviewer’s positive comments.**

Reviewer #3: Adequate.

**[Response] We appreciate the reviewer’s positive comments.**

**Results**

Reviewer #2: The results are clear and correctly presented, as are the tables and figures.

**[Response] Many thanks for the reviewer’s positive comments.**

Reviewer #3: Adequate.

**[Response] We appreciate the reviewer’s positive comments.**

**Conclusions**

Reviewer #2: The conclusions are supported by the results.

Study limitations are presented.

**[Response]** **Many thanks for the reviewer’s positive comments on our manuscript.**

Reviewer #3: Adequate.

**[Response] We appreciate the reviewer’s positive comments.**

**Editorial and Data Presentation Modifications**

Reviewer #2: Accept

**[Response] Many thanks for the reviewer’s positive comments.**

Reviewer #3: Accept after minor corrections.

**[Response]** **Many thanks for the reviewer’s helpful corrections. Done as suggested.**

**Summary and General Comments**

Reviewer #2: The manuscript entitled “Human infection with respiratory adenovirus in China: a systematic review and meta-analysis”, authored by Mei-Chen Liu et al, described the epidemiological and clinical features of HAdV infections in China, from January 2009 to March 2021. In addition, the genetic and epidemiological characteristics of HAdVs were investigated.

I want to emphasize that the work is very well written and concisely covers all the epidemiological characteristics of HAdV respiratory infections.

The work carried out is a very important contribution to the knowledge of the classical and molecular epidemiology of HAdV in China and also provides valuable information worldwide.

**[Response] Many thanks for the reviewer’s positive comments.**

Reviewer #3: The authors made changes to the manuscript to address the reviewers' comments. A few corrections are still necessary before publication, as pointed in the pdf file (attached).

**[Response]** **We appreciate the reviewer’s positive comments and helpful corrections. We have made these corrections in the manuscript and checked for similar problems, including two grammar issues (Page 5, Lines 66 and 80), singular and plural issues (Page 16, Line 269; Page 23, Line 411), and the missing space before the reference number throughout the main text.**
